# Growth of candidate phyla radiation bacteria in groundwater incubations reveals widespread adaptations to oxic conditions

**DOI:** 10.1186/s40168-025-02244-1

**Published:** 2025-10-30

**Authors:** Ekaterine Gabashvili, Kirsten Küsel, Akbar Adjie Pratama, He Wang, Martin Taubert

**Affiliations:** 1 Aquatic Geomicrobiology, Institute of Biodiversity, Ecology & Evolution, Friedrich Schiller University Jena, Dornburger Str. 159, Jena, 07743 Germany; 2 Cluster of Excellence Balance of the Microverse, Friedrich Schiller University Jena, Jena, Germany; 3 German Center for Integrative Biodiversity Research (iDiv) Halle-Jena-Leipzig, Puschstrasse 4, Leipzig, 04103 Germany; 4 Department of Microbiology, Ohio State University, Columbus, OH 43210 USA; 5 Center of Microbiome Science, The Ohio State University, Columbus, OH USA; 6 National Science Foundation EMERGE Biology Integration Institute, Columbus, OH USA

**Keywords:** Candidate phyla radiation, Patescibacteria, Groundwater, Autotrophy, Methylotrophy

## Abstract

**Background:**

The candidate phyla radiation (CPR) comprises a widespread but poorly understood group of bacteria with limited cultured representatives, largely due to their metabolic dependencies on microbial hosts. In laboratory incubations, CPR often decline sharply in relative abundance, even when samples originate from natural environments where they dominate, such as groundwater, where they can represent over 50% of the microbiome. Suitable enrichment conditions and host interactions remain poorly defined.

**Results:**

Here, we analyzed 16S rRNA gene amplicon data from 397 groundwater incubation samples across 31 treatments, including 22 under oxic conditions, to identify factors that promote CPR survival and growth. Despite an initial decline, CPR abundances recovered over longer incubation times, reaching up to 11–30% of the microbial community. In total, we detected 1410 CPR amplicon sequence variants (ASVs), spanning six major CPR classes commonly found in groundwater. Enrichment success was treatment-specific: *Cand.* Saccharimonadia dominated in incubations with polysaccharides (up to 31.4%), while *Cand.* Parcubacteria were enriched (> 23%) in treatments stimulating methylotrophs and autotrophs. ASV-specific growth rates based on quantitative PCR showed that some CPR doubled within 1–2 days, comparable to faster-growing non-CPR groundwater bacteria, while most CPR had doubling times around 15 days.

Strikingly, although the relative abundance of many CPR ASVs showed positive correlation with anoxic conditions, overall CPR reached higher absolute abundances under oxic conditions than under anoxic conditions. Metabolic network analysis based on metagenome-assembled genomes revealed that up to 62% of annotated genes were associated with functions linked to oxic conditions. In fact, 25 CPR genomes encoded enzymes that directly utilize oxygen, challenging the long-standing view of CPR as strictly anaerobic, fermentative organisms.

**Conclusions:**

Our findings demonstrate that diverse CPR lineages not only survive but actively grow in groundwater incubations, even under oxic conditions. The discovery of genes for oxygen-dependent reactions and substantial CPR enrichment in oxic treatments reveals unexpected metabolic flexibility, helping to explain their persistence and ecological success across a wide range of environments.

**Supplementary Information:**

The online version contains supplementary material available at 10.1186/s40168-025-02244-1.

## Introduction

The candidate phyla radiation (CPR) constitutes a recently uncovered, expansive bacterial group, primarily investigated through metagenomic studies [1, 2]. Although its exact phylogenetic placement as one or multiple phyla is still debated, it represents a large fraction of bacterial diversity [3–6]. CPR appear ubiquitously in a variety of environments, including freshwater [7, 8] and marine ecosystems [9], as well as animal-associated habitats [10]. In particular, in oligotrophic subsurface habitats like groundwater, they can account for up to 50% of the microbial community [11–13]. Their high abundance points to a potentially crucial role within groundwater microbiomes, yet their ecological functions remain largely unresolved. 

CPR are typically characterized by ultra-small cell sizes of 100 to 200 nm in diameter [6, 7, 14]. They feature significantly reduced genomes under 1 Mbp [8], which exhibit a severely limited metabolic potential. CPR primarily seem to rely on anaerobic, fermentative metabolism as they mostly lack the genes necessary for respiration, while their genomes often encode fermentative pathways [5, 15, 16]. Additionally, they encode carbohydrate-active enzymes, which likely play a key role in transforming precursors for fermentation [17]. Despite this anaerobic lifestyle, interestingly CPR are also often present in oxic environments, and have been shown to possess functions for oxidative stress tolerance [11, 18].

In addition, the streamlined genomes of CPR often even lack the metabolic capability to synthesize essential cellular components such as nucleotides, amino acids, and lipids [19]. It has been hypothesized that CPR hence employ an external source of these compounds. Several studies suggest that many CPR are host-associated, engaging in episymbiotic relationships with other microorganisms [10, 20–22]. This, in combination with the overall reduced genome and cell sizes, likely represents an adaptation to low nutrient availability, fitting for energy-efficient, slow growth in oligotrophic habitats. A parasitic lifestyle would underscore the impact of CPR on microbial communities; however, this host-dependence complicates cultivation attempts. So far, only a few lineages, including *Cand.* Saccharimonadia and *Cand.* Absconditabacteria, have been successfully isolated in binary cultures with their hosts, facilitated by their episymbiotic lifestyle [5, 6]. Organisms from some CPR clades (e.g., ABY1, *Cand.* Paceibacteria/Parcubacteria, *Cand.* Saccharimonadia) also appear to be free-living [8] despite the reliance on compounds produced by other organisms, but so far, no free-living CPR have been isolated. Obtaining CPR in cultures or enrichments, however, is necessary for an assessment of their functions.

In the groundwater of the Hainich Critical Zone Exploratory (CZE), located in western Thuringia in Germany [23], a wide variety of CPR, including *Cand.* Parcubacteria, clade ABY, *Cand.* Gracillibacteria, and *Cand.* Saccharimonadia, thrive under a gradient from oxic to anoxic conditions [11]. This unique setting has been the basis for numerous incubation experiments aimed at identifying key environmental drivers and metabolic processes within the groundwater microbiome. Previous experiments predominantly focused on organic carbon degradation and primary production, often under conditions favoring fast-growing, non-CPR taxa, which likely outcompeted the slower-growing CPR populations [24–28]. Despite this bias toward non-CPR taxa, hints of CPR enrichment emerged, motivating a systematic investigation of the conditions enabling their persistence and growth.

Here, we take a systematic and comprehensive approach to investigate the occurrence of CPR across these experiments. Based on amplicon sequencing variants (ASVs) from 16S rRNA gene amplicon sequencing and quantitative PCR data from 397 samples representing 31 different treatments, including 22 under oxic conditions, which extended up to 3 years, we identified patterns of CPR enrichment across varying incubation regimes. By leveraging this extensive dataset, our goal was to determine which CPR lineages were capable of sustained growth and to define the environmental conditions most suitable for their enrichment.

To further understand the ecological potential of CPR in oxic environments, we linked our observations from the incubation experiments with metagenome-assembled genomes (MAGs) obtained in a previous study [11]. This integration allowed us to identify genetic features within specific CPR clades that may underpin their ability to grow under oxic conditions. In doing so, our study not only reveals favorable enrichment conditions for groundwater CPR in laboratory settings, but also provides new perspectives on their ecological roles and genomic signatures of adaptation to oxic environments.

## Methods

### Overview of groundwater incubations

For investigating the dynamics of the CPR community in groundwater incubations, a comprehensive bacterial 16S rRNA amplicon sequencing dataset of previously conducted incubation experiments was used, including 22 treatments under oxic and 9 experiments under anoxic conditions, with 397 samples in total (Table S1). All incubations were conducted in oligotrophic groundwater with minimal supplements, avoiding the use of rich media, vitamins, or trace elements. The incubation experiments were originally used to investigate a range of different microbial processes or to enrich certain functional key players, and were therefore prepared employing slightly different methods. Eleven of the treatments, with 107 samples, have been included in previous publications [24–28]. The remaining treatments, with 290 samples, in particular from long-term enrichments of more than 1 year, represent novel data, and detailed information for specific experiments can be found in the Supplementary Information. Below, a generic description of the setup of the incubation experiments is given.

### Sampling site

Groundwater was collected from two wells of the groundwater monitoring transect of the Hainich CZE [23]. Well H41 (51.1150842N, 10.4479713E) accesses an aquifer assemblage at 48 m depth in a trochite limestone stratum. Groundwater of this well is sourced by a beech forest (*Fagus sylvatica*) recharge area and features mean dissolved oxygen concentrations of 5.0 ± 1.55 mg L^−1^, < 0.1 mg L^−1^ ammonium, 1.9 ± 1.5 mg L^−1^ dissolved organic carbon, 70.8 ± 12.7 mg L^−1^ total inorganic carbon, and a pH of 7.2 [29, 30]. Well H52 accesses an aquifer assemblage in the Meissner formation of the Upper Muschelkalk (Middle Triassic), dominated by alternating mudstone and limestone, at 65 m depth. The groundwater is characterized by anoxic conditions, 1.0 ± 4.0 mg L^−1^ nitrate, 90.0 ± 9.9 mg L^−1^ sulfate, around 1 mg L^−1^ dissolved organic carbon, 85.1 ± 6.0 mg L^−1^ total inorganic carbon and a pH of 7.3 ± 0.1 [31].

### Setup and monitoring of incubation experiments

Groundwater for the incubation experiments was sampled using a submersible pump (Grundfos MP1, Grundfos, Bjerringbro, Denmark). Due to the low cell numbers in the oligotrophic groundwater, different strategies were used to accumulate sufficient biomass for later analyses. As one strategy, biomass was enriched before setup of the experiment by filtration through 0.3 µm pore size glass fiber filters or 0.2 µm pore size polyethersulfone membrane supor® filters (PALL Corporation, Michigan, USA) and parts of these filters were then included in the incubations, as previously described [28]. As another strategy, incubation volumes were scaled up, to up to 10 L, and biomass was collected after incubation by filtration on supor® filters as described above.

In incubations with groundwater from well H41, oxic conditions were ensured by adding SP-PSt3-YAU-D3-YOP sensor spots (PreSens, Regensburg, Germany) to the incubation bottles, allowing noninvasive monitoring of oxygen concentrations with a Fibox 4 detector (PreSens). Bottles were opened for aeration if oxygen concentrations dropped below 100 µmol/L. For incubations with groundwater from well H52, anoxic conditions were ensured by flushing the incubation bottles with N_2_ directly after sampling and bottling of the groundwater, as well as monitoring potential oxygen input with the sensor spots described above.

All incubations were carried out in glass bottles with butyl rubber stoppers. Different supplements were added to selectively promote certain microbial processes (see Table S1 for details). Individual incubations were carried out for several days to several years (see Table S1 for details) with rotational shaking (100 rpm) at 15 °C in the dark.

### Classification of experimental treatments in the manuscript

Based on the microbial processes stimulated by the conditions used in each treatment, these were subdivided into four groups: “Auto” treatments aiming to stimulate chemolithoautotrophic growth featured inorganic electron donors like thiosulfate, ammonium, and nitrite as supplements. “Methylo” treatments were supplemented with methylamine or methanol to stimulate methylotrophy. “Defined” treatments included chemically defined organic compounds like veratric acid, cellulose, or starch, and “Complex” treatments contained complex mixtures of organic compounds such as leaf leachate, soil seepage, microbial necromass, or R2A medium containing yeast extract and peptone [32].

In addition to these four treatments, for some experiments also samples before incubation were collected. Owing to the logistics of sampling and transporting groundwater from the field site to the lab, incubation experiments were typically started several days after the groundwater was pumped from the wells. To differentiate between samples from these two time points, samples obtained directly from the field are named “In situ,” while samples from the start of the incubation experiments some time later are named “start” throughout the manuscript.

### 16S rRNA amplicon sequencing from enrichment experiment

After incubation, the biomass was collected on 0.2 µm filters, and DNA extraction was conducted as previously described [28]. The V3 to V5 region of bacterial 16S rRNA genes were amplified with primer pair Bakt341F (5′-CCT ACG GGN GGC WGC AG-3′) and Bakt785R (5′-GAC TAC HVG GGT ATC TAA TCC-3′) [33] and HotStarTaq Mastermix (QIAGEN, Hilden, Germany) as previously described [34]. Illumina sequencing of the amplicons was carried out in-house on a MiSeq platform (Illumina, Eindhoven, The Netherlands) with v3 chemistry as previously described [34].

### DADA2 analysis

The raw 16S rRNA gene sequences from all samples were analyzed collectively using the DADA2 pipeline (version 1.26.0), implemented in the R software environment (version 4.2.2) [35]. During this analysis, the sequences were first quality-filtered: The packages ShortRead (version 1.56.1) [36] and Biostrings (version 2.66.0) [37] were used to confirm that forward reads start with the forward primer and reverse reads start with the reverse primer. Subsequently, primers were trimmed with fixed length (17 nt for forward, 21 nt for reverse reads, i.e., the primer lengths) using the filterAndTrim() function, with additional parameters truncQ = 2 (truncates read when nt quality score is 2 or lower), maxEE = c(2,2) (removes reads with more than 2 expected errors) and maxN = 0 (only keeps reads without ambiguous bases). Paired-end reads were merged and amplicon sequence variants (ASVs) were generated, followed by removal of chimeras using the removeBimeraDenovo() function with method = “consensus”. The taxonomy of the ASVs was assigned based on the SILVA reference database (version 138.1) [38]. The ASVs read counts for each sample were normalized using total sum scaling, resulting in relative abundances for each ASV.

### Estimation of absolute abundances and growth rates

For Auto and Methylo treatments, total bacterial 16S rRNA gene copy numbers were determined by quantitative PCR using the primer combination Bac8Fmod (5′-AGAGTTTGATYMTGGCTCAG-3′) [39] and Bac338Rabc (5′-GCW GCC WCC CGT AGG WGT-3′) [40]. Quantitative PCR was conducted using the Brilliant II SYBR Green QPCR Mastermix (Agilent Technologies, Waldbronn, Germany) on a Mx3000P cycler (Agilent Technologies) as previously described [41]. Absolute abundance of each ASV was calculated by multiplying the total bacterial 16S rRNA gene copy numbers and the corresponding relative abundance from high-throughput sequencing. Based on the estimated absolute gene copy numbers (N) per ASV (i), growth rates (k) during the incubation time (t) of enrichment experiments were calculated according to the exponential growth Eq. (1).

1 $${N}_{i}\left(t\right)={N}_{i}\left(0\right){e}^{kt}$$

To facilitate calculation, linear regression analysis was employed based on the incubation time and the natural logarithm of the absolute gene copy numbers to calculate growth rates for each ASV in each experiment where this ASV occurred at least at three time points. The slopes obtained from the linear regression analysis resulted in the desired growth rates. Negative growth rates were discarded, and from the remaining growth rates, doubling times (T_d_) were derived using formula (2).

2 $${T}_{d}=\text{ln}2/k$$

The estimated growth rates assume stable growth over the observation period, which was likely not the case in the dynamic conditions of the groundwater enrichments. In reality, organisms may have grown substantially faster during certain periods and slower during others, depending on shifts in nutrient availability or microbial interactions. Nevertheless, the calculated values represent the minimum growth rates required to account for the observed increases in abundance. As such, they provide conservative estimates, while actual peak growth rates may have been considerably higher.

### Assessment of primer coverage

To determine whether our analysis was able to capture the high diversity of the CPR, the coverage of the primer pair Bakt341F/Bakt785R [33] used for amplicon sequencing and the primer pair Bac8Fmod/Bac338Rabc [39, 40] used for quantitative PCR was assessed. An in silico analysis was conducted using the SILVA SSU r138.2 database, allowing up to two mismatches and requiring a two-base perfect match at the 3′ end to reflect realistic PCR conditions. The results indicated a coverage of the primer pair Bakt341F/Bakt785R above 90% for all investigated CPR classes (see Table S2), with the exception of *Cand.* Microgenomatia, which are not covered by this primer pair. The primer pair Bac8Fmod/Bac338Rabc covered all six detected CPR classes, including *Cand.* Microgenomatia, to more than 80%.

### Co-occurrence network analysis

The ecological associations and statistical inferences among different taxa and CPR were evaluated at the ASV level using bacterial relative abundance data. Sparcc analysis in R package SpiecEasi (version 1.1.2) [42] was employed to infer networks among the ASVs of CPR with non-CPR taxa, individually within each treatment. The edge lists of the networks were retrieved using the R igraph package (version 1.5.0.1) [43]. Only edge lists featuring CPR, with a positive correlation, and an edge weight exceeding 0.1 were considered. Subsequently, the numbers of these edge lists connecting each CPR order with each non-CPR order were counted per treatment. Chord diagrams for visualization of connections between CPR and non-CPR were generated using the circlize package (version 0.4.16) [44].

### Correlation of CPR ASVs with supplements

To assess associations between CPR ASVs and incubation parameters (supplements and oxic/anoxic conditions), we first applied differential abundance testing with ANCOM-BC2 (R package *ANCOMBC*, function ancombc2) [45]. ASVs with adjusted *p* values < 0.05 were considered significant, and log2-fold changes were used to determine the direction of association. However, ANCOM-BC does not return results for parameters present in only a small number of samples. To capture potential associations with such rare conditions, we additionally calculated Spearman rank correlations between supplement presence/absence and relative ASV abundances. Given that correlation analyses on compositional data are less robust, we applied a more stringent significance cutoff (*p* < 0.001). Significant positive and negative correlations were visualized using the *ComplexHeatmap* package (version 2.14.0) [46]. We note that correlations observed in these cases may also reflect co-varying factors and should therefore be interpreted with caution.

### Oxygen-related metabolic potential of CPR

To determine putative metabolic functions in CPR related to oxygen, previously published metagenome-assembled genomes (MAGs) of CPR from groundwater of the Hainich CZE [11], from the same aquifers where samples for ASV generation originated, were employed. This dataset contained 587 CPR-MAGs with ≥ 50% completeness and ≤ 10% contamination. Functional annotation of genes in the MAGs was conducted with DRAM [47] and METABOLIC-G [48], using default settings. In a first analysis, the resulting annotations were mapped to an oxic/anoxic metabolic reaction network based on EC numbers, as previously described [49, 50], to identify oxygen-dependent reactions. EC numbers were extracted for each annotated gene from the MAGs and summarized per MAG, without counting duplicates, leveraging that the KEGG-based annotations typically included the respective EC numbers describing the reaction(s) catalyzed by the gene products. Genes annotated with more than one EC number, representing only a small fraction of the dataset, were excluded from this analysis. The resulting set of EC numbers was compared against the reaction network database to obtain counts of oxygen-dependent and oxygen-independent reactions per MAG. For reactions matching the oxic subnetwork, we used the corresponding KEGG Orthologies from the original annotations to perform a more detailed investigation across the CPR.

As a second, complementary analysis, we focused on the genetic potential of two groups of CPR found to be preferentially enriched under oxic conditions, *Cand.* Saccharimonadia and *Cand.* Berkelbacteria (UBA1384). The metabolic functions encoded in the genomes of these organisms were compared to those of all other CPR taxa combined. To identify functions with significantly different distributions, Kruskal–Wallis test followed by Dunn’s post-hoc test with Bonferroni correction was conducted, and functions resulting in *p* values below 0.05 were selected.

### Statistical analysis and figure generation

Statistical tests were conducted in R using the Vegan package (v2.6–4) [51] along with tidyverse (v1.3.1) [52]. Diversity of the CPR community per sample was determined by filtering for CPR ASVs and calculating the Shannon Diversity index using the diversity() function. Figures were generated with ggplot2 (v3.5.1) [53] including ggrepel (v0.9.5) [54], unless otherwise described. R code used for analyses and figure generation has been deposited in github (https://github.com/m-taubert/cpr_groundwater_enrichments).

## Results

### CPR are maintained and grow in groundwater incubations

**Fig. 1:**
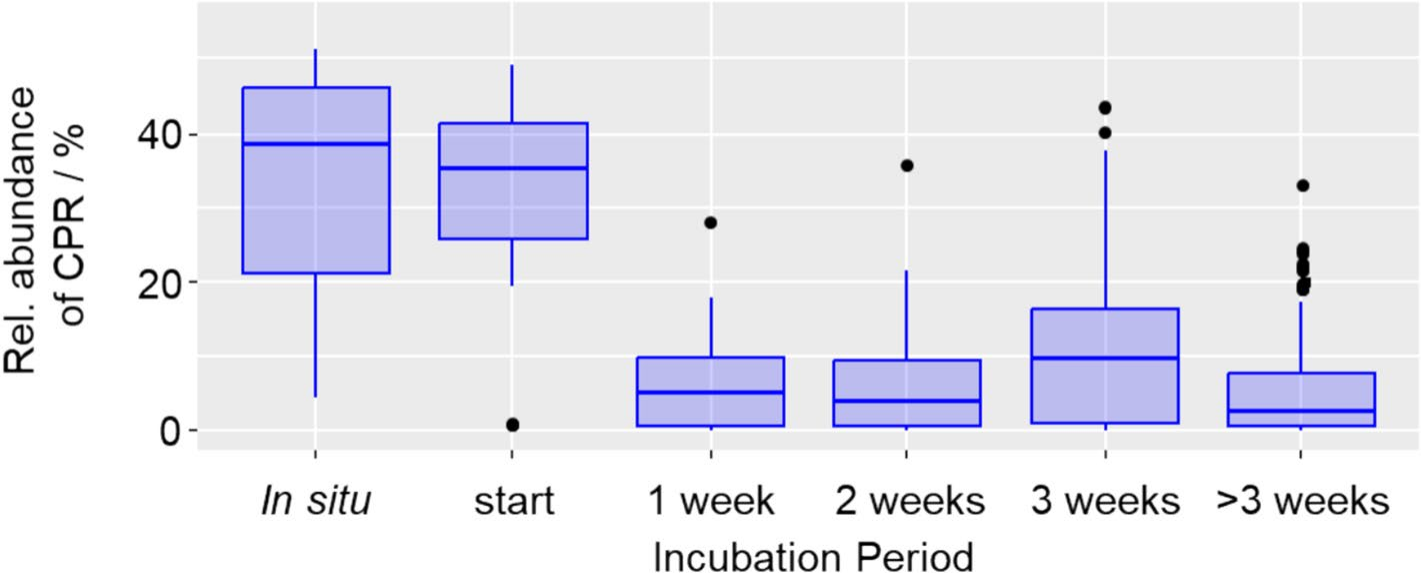
Relative abundances of CPR in incubation experiments. Shown are relative abundances of CPR based on 16S rRNA gene amplicon sequencing summarized across all treatments. In situ data was obtained from groundwater filtered directly after sampling, and start data directly after setting up groundwater incubations. Boxes show median, first and third quartile, whiskers show minimum and maximum values, excluding outliers defined as exceeding 1.5 * interquartile range, which are shown as points

All groundwater incubation experiments showed a drastic decline of the relative abundance of CPR, from up to 51.3% in situ down to 6.4% within 1 week of incubation (Fig. 1A). During prolonged incubation, relative abundances typically remained in the range of 4.7–12.0%. Individual samples, however, showed higher enrichment of CPR above 15%, especially after more than a year of incubation (Fig. S1), coming back into the range observed in situ.

Quantitative PCR data of total bacterial 16S rRNA gene copies, only available for the Auto and Methylo treatments, likewise showed an initial decrease from 1.0 × 10^8^ (median; IQR: 3.6 × 10^7^–5.4 × 10^8^) gene copies per liter to 5.8 × 10^6^ (IQR: 8.2 × 10^6^–4.1 × 10^7^) after setup. Subsequently, gene copy numbers increased again to 1.1 × 10^9^ (IRQ: 4.6 × 10^7^–4.3 × 10^9^) per liter, reaching significantly higher levels than in situ (Dunn’s post-hoc test, p_Auto_ = 0.011, p_Methylo_ = 0.0001) (Fig. 1B). This increase was mainly caused by the growth of phyla like Proteobacteria, Bacteroidota, Planctomycetota, and Verrucomicrobiota, stimulated in the different treatments due to supplement addition as indicated by their increase in relative abundance (Fig. S2).

By combining relative CPR abundances and total 16S rRNA gene copy numbers in these treatments, we estimated the absolute abundances of CPR 16S gene copies. In the Auto treatments supplemented with inorganic electron donors, estimated CPR gene copies showed an increase up to two orders of magnitude during the first 2 weeks of incubations, reaching values of 1.7 × 10^8^ (IQR: 5.4 × 10^7^–1.4 × 10^9^) per liter, with a subsequent decline (Fig. 1C). The peak in CPR abundance after 2 weeks was even significantly higher than the CPR gene copy numbers observed in situ, with estimates of 3.7 × 10^7^ (IQR: 6.6 × 10^6^–9.6 × 10^7^) per liter (Dunn’s post-hoc test, *p* = 3.0 × 10^–9^). For the Methylo treatments supplemented with reduced C_1_ compounds, absolute CPR gene copies increased over a longer period, reaching the highest values of 1.1 × 10^7^ (IQR: 7.6 × 10^6^–4.8 × 10^7^) after more than 3 weeks of incubation. These results show that following prolonged incubation, absolute abundances of CPR could reach levels similar to or even exceeding those observed in situ.

### Groundwater incubations sustained a diverse range of CPR taxa

**Fig. 2:**
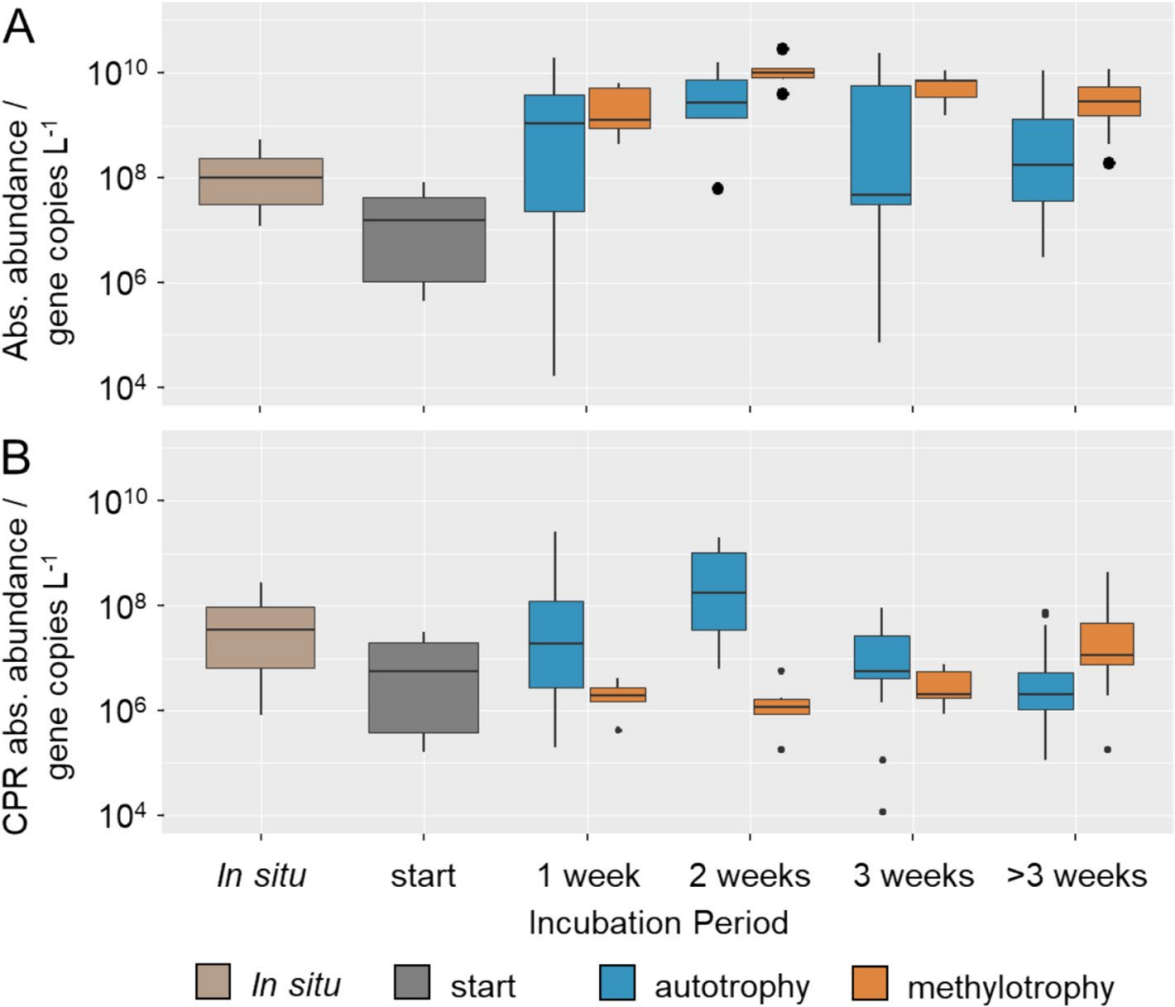
Absolute abundances of CPR in incubation experiments. Shown are **A** the absolute abundances of bacterial 16S rRNA gene copies based on quantitative PCR, and **B** estimates for the absolute abundances of CPR 16S rRNA gene copies derived from multiplying absolute bacterial 16S rRNA gene copies and relative CPR abundances. In situ data was obtained from groundwater filtered directly after sampling, and start data directly after setting up groundwater incubations. Only Auto and Methylo treatments where qPCR data was available are included. Boxes show median, first and third quartile, whiskers show minimum and maximum values, excluding outliers defined as exceeding 1.5 * interquartile range, which are shown as points

Across all treatments and time points, the groundwater incubations harbored a large diversity of CPR taxa, represented by a total of 1410 ASVs. The majority of these were affiliated with the CPR classes *Cand.* Parcubacteria, with 791 ASVs, and ABY1, with 339 ASVs. Furthermore, *Cand.* Gracilibacteria (165 ASVs), *Cand.* Saccharimonadia (71 ASVs), *Cand.* Berkelbacteria (30 ASVs), and *Cand.* Microgenomatia (3 ASVs) were detected. On order level, the most diverse taxa were *Cand.* Magasanikbacteria (ABY1) with 162 ASVs, as well as *Cand.* Kaiserbacteria and UBA9983 (*Cand.* Parcubacteria) with 105 and 44 ASVs, respectively (Fig. 2).

**Fig. 3:**
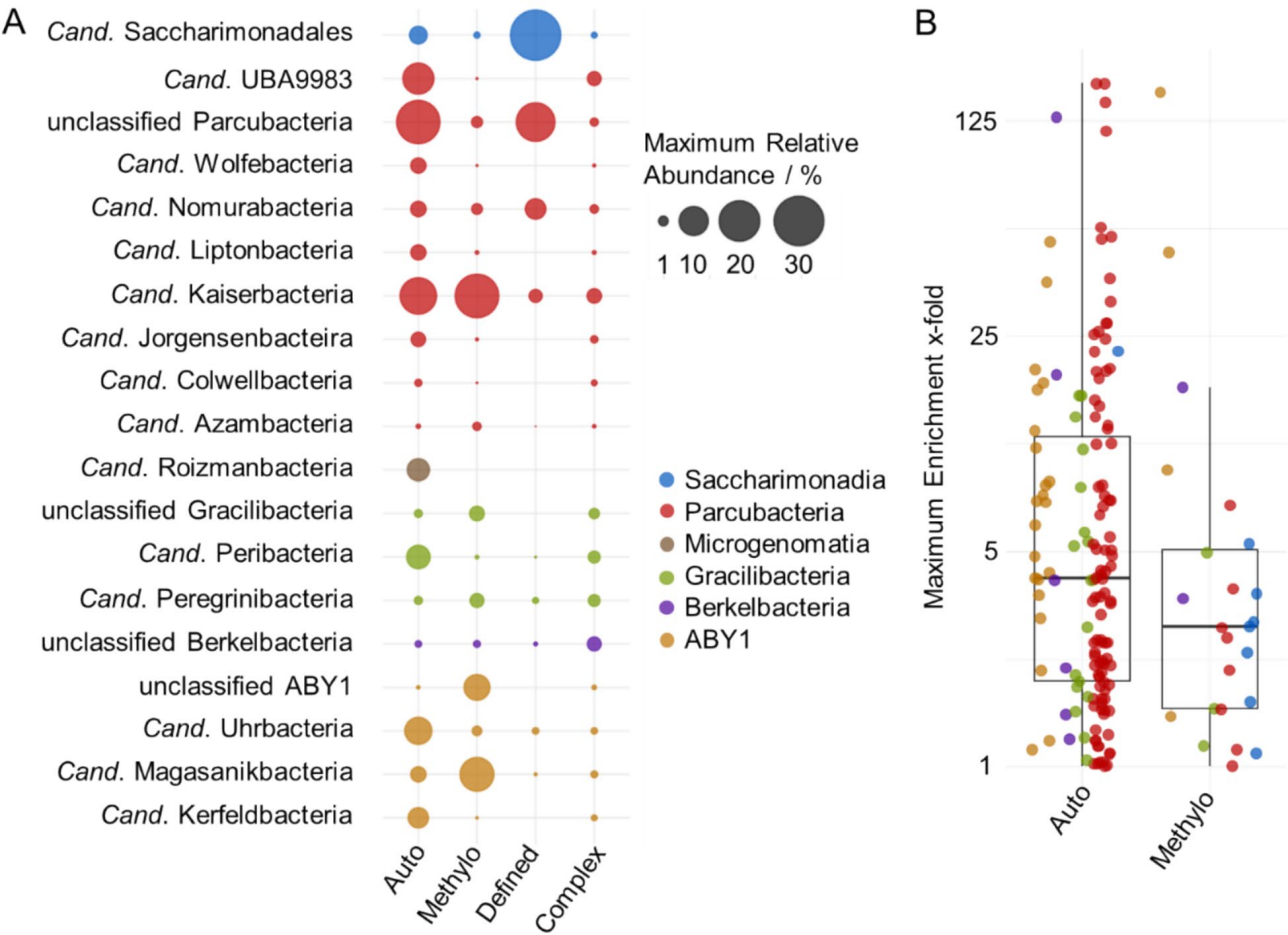
Enrichment of CPR taxa in incubation experiments. **A** Maximal relative abundance of CPR orders across treatments based on 16S rRNA gene sequencing. **B** Maximum fold absolute enrichment (log scale) of ASVs compared to the start of the incubations, based on 16S rRNA gene sequencing and quantitative PCR. Only ASVs with increases are shown. Boxes show median, first and third quartile, whiskers show minimum and maximum values, excluding outliers defined as exceeding 1.5 * interquartile range, which are shown as points

The presence of these CPR taxa was strongly influenced by the treatments. The Auto treatments supported the highest Shannon diversity of CPR (Fig. S3), featuring ASVs from *Cand.* Parcubacteria (up to 23.2% relative abundance), ABY1 (8.8%), *Cand.* Gracilibacteria (6.5%), *Cand.* Microgenomatia (5.7%), and *Cand.* Saccharimonadia (3.2%) (Fig. 3A). The Methylo treatments contained mainly ABY1 and *Cand.* Parcubacteria, with up to 13.9% and 23.2% relative abundance, respectively. The major orders in these treatments were *Cand.* Kaiserbacteria, *Cand.* Nomurabacteria, and UBA9983 (*Cand.* Parcubacteria) as well as *Cand.* Magasanikbacteria (ABY1). In the Defined treatments supplemented with chemically defined organic compounds, *Cand.* Saccharimonadia were dominant, with up to 31.4% relative abundance. *Cand.* Parcubacteria were also present with relative abundance up to 18.4%. The Complex treatments supplemented with complex mixtures of organic compounds included various CPR groups, though at low relative abundances of less than 2.2% each.

We further quantified the absolute enrichments of individual ASVs in the Auto and Methylo treatments based on qPCR data, and observed that 42 ASVs reached more than tenfold enrichment compared to the start of the incubations, and 6 ASVs even reached more than 100-fold enrichment (Fig. 3B). These ASVs were mostly affiliated with *Cand.* Parcubacteria, such as group UBA9983 and *Cand.* Kaiserbacteria, in the Auto treatments, and with ABY1, in particular *Cand.* Magasanikbacteria, in the Methylo treatments. These results show a specific response of CPR taxa and ASVs to incubation conditions across treatments.

### Distinct CPR ASVs were enriched by added supplements

**Fig. 4:**
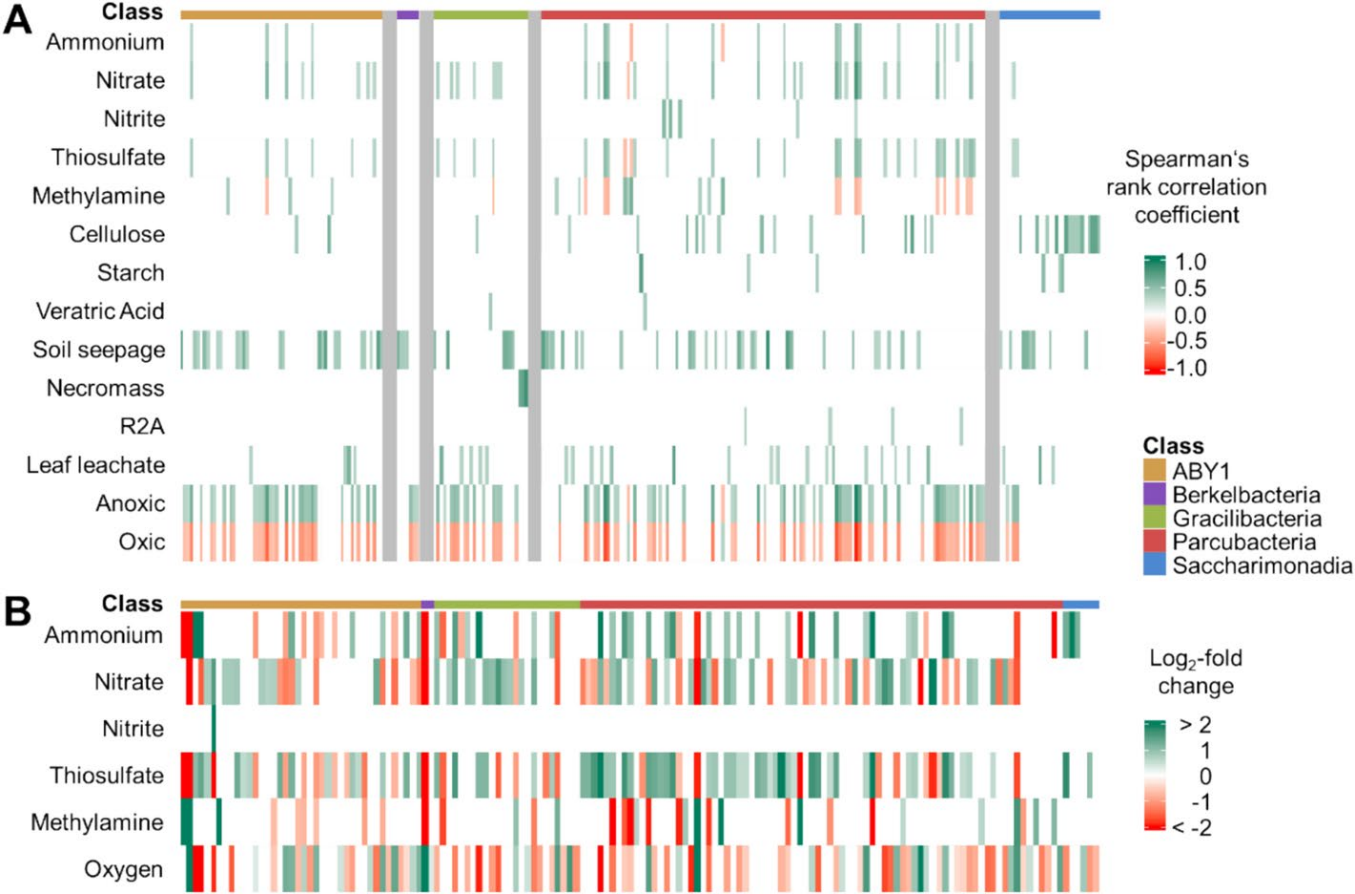
Correlations of CPR ASVs with supplements and incubation conditions. Columns correspond to individual ASVs. **A** Results from Spearman’s correlation analysis, showing 265 ASVs with significant correlations (*p* > 0.001) out of the total of 1410 ASVs (see Table S3). Green shades indicate positive and red shades negative Spearman’s rank correlation coefficients, respectively. **B** Results from differential abundance testing with ANCOM-BC, showing 152 ASVs with significant changes of abundance out of the total of 1410 ASVs (see Table S4). Green shades indicate positive and red shades negative log_2_-fold changes in abundance

Supplementation of groundwater incubations with inorganic electron donors, soil seepage, or cellulose resulted in an enrichment of the highest number of CPR ASVs. Nitrate and thiosulfate addition correlated with 44 ASVs across CPR groups, with strongest correlations (R^2^: 0.4–0.65) observed for 14 ASVs of *Cand.* Parcubacteria based on Spearman’s correlation analysis (Fig. 3A). Differential abundance testing indicated enrichment of 61 ASVs with thiosulfate, 48 ASVs with nitrate and 36 ASVs with ammonium. Also here, most ASVs were affiliated with *Cand.* Parcubacteria (51 ASVs) and ABY1 (22 ASVs) (Fig. 4B). Addition of soil seepage, i.e., water moving through the soils sampled with lysimeters, correlated with the relative abundances of a diverse range of *Cand.* Parcubateria (18 ASVs), ABY1 (10 ASVs), *Cand.* Gracilibacteria (5 ASVs), *Cand.* Saccharimonadales (5 ASVs), and *Cand.* Berkelbacteria (2 ASVs), based on Spearman’s correlation analysis (Fig. 4A). Furthermore, cellulose correlated with 31 ASVs, mostly from *Cand.* Saccharimonadia and *Cand.* Parcubacteria, while starch only correlated with six ASVs from these groups.

Other supplements showed strong correlations only to a low number of ASVs: microbial necromass addition showed the strongest correlations observed, R^2^ values of 0.67–0.85, with 3 ASVs of *Cand.* Gracilibacteria. Methylamine correlated strongly positively with 3 ASVs of *Cand.* Kaiserbacteria (*Cand.* Parcubacteria), and less strongly with 3 ASVs of *Cand.* Magasanikbacteria (ABY1), while also showing negative correlations with several ASVs based on Spearman’s correlation analysis. Also differential abundance testing showed 15 ASVs with positive and 19 ASVs with negative log2-fold changes on methylamine (Fig. 4B).

The highest effects were observed when comparing anoxic and oxic incubations. A total of 102 ASVs showed positive correlations with anoxic conditions, while only 2 ASVs, belonging to *Cand.* Parcubacteria, correlated positively with oxic conditions based on Spearman’s correlation analysis (Fig. 4A). Differential abundance testing also revealed a higher number of ASVs enriched without oxygen (58 ASVs) than with oxygen (34 ASVs). This indicated that a higher number of CPR showed a significant preference for growth under anoxic conditions than oxic conditions. However, despite this trend, a considerable number of ASVs were found exclusively under oxic conditions, suggesting that many CPR may still favor or tolerate oxic environments. Specifically, 472 ASVs were found exclusively under anoxic conditions, and 343 ASVs were found only under oxic conditions, with 79 ASVs shared between both. This highlights that while anoxic conditions favor more CPR taxa in terms of statistically significant correlations, both conditions support distinct CPR communities.

### Growth rates of CPR match those of other groundwater bacteria

**Fig. 5:**
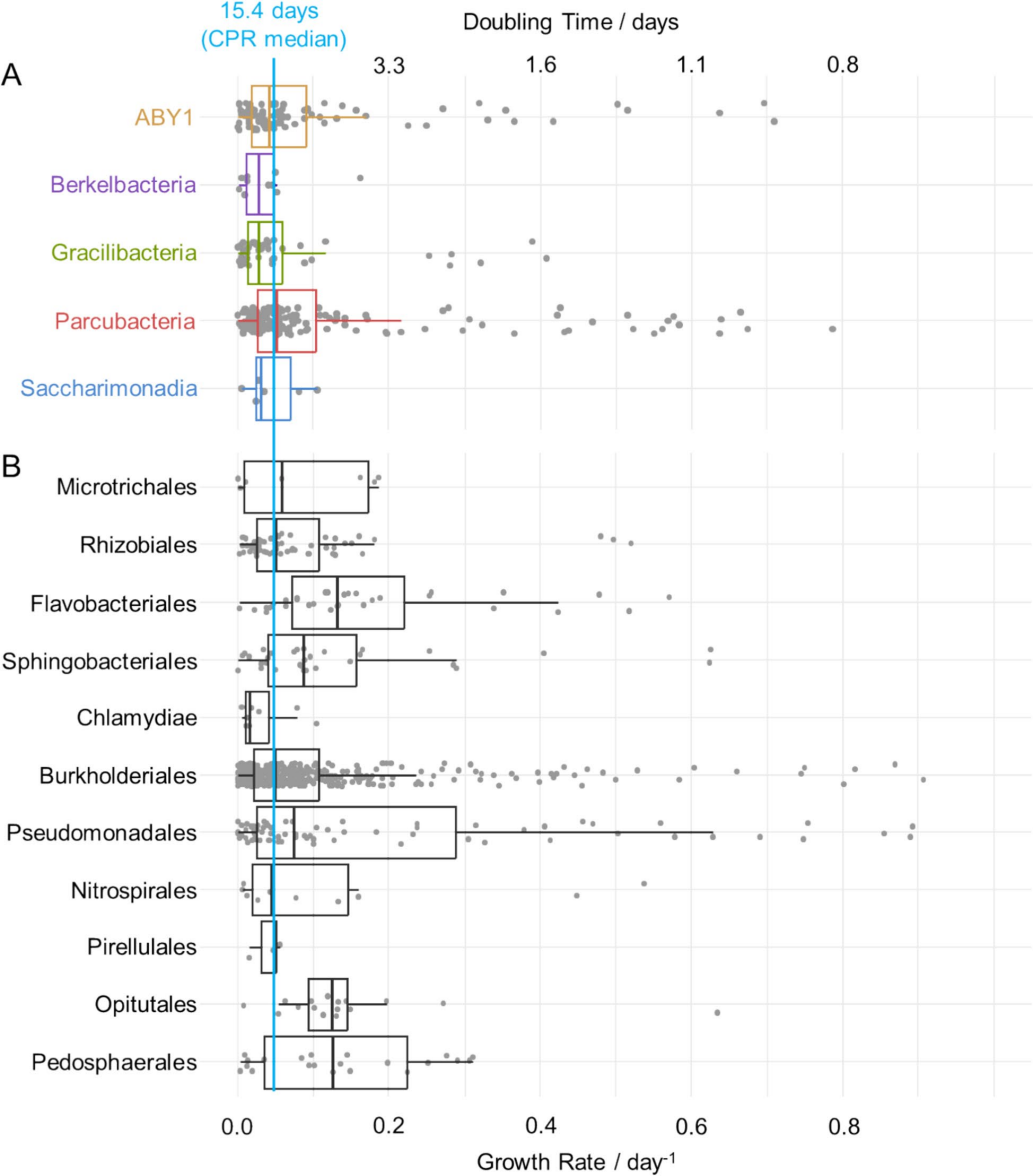
Doubling times (top axis) and growth rates (bottom axis) of ASVs in enrichments. Shown are values for **A** CPR ASVs and **B** non-CPR ASVs based on 16S rRNA gene sequencing and quantitative PCR. Only ASVs occurring on at least 3 time points in an experiment and with a positive growth rate are shown, and only experiments with available qPCR data from Auto and Methylo treatments are included. Boxes show median, first and third quartile, whiskers show minimum and maximum values

When estimating doubling times for CPR and non-CPR, we observed a broad range for both groups. The majority of CPR ASVs showing growth during incubations exhibited doubling times around 15 days, but surprisingly, some ASVs were able to double within as little as 1 to 2 days (Fig. 5A). These fast-growing CPR included 20 ASVs of *Cand.* Parcubacteria, affiliated with UBA9983, *Cand.* Kaiserbacteria, *Cand.* Lloydbacteria, *Cand.* Nomurabacteria, *Cand.* Jorgensenbacteria, and *Cand.* Wolfebacteria. Furthermore, members of ABY1 (10 ASVs) mainly affiliated with *Cand.* Magasanikbacteria, *Cand.* Uhrbacteria, and *Cand.* Kerfeldbacteria, and 3 ASVs of *Cand.* Gracilibacteria (*Cand.* Peregrinibacteria) exhibited doubling times below 2 days. The faster-growing members of *Cand.* Saccharimonadia and *Cand.* Berkelbacteria only reached doubling times of 6 and 4 days, respectively.

Differences in growth were also observed between the slower-growing CPRs across classes. While *Cand.* Parcubacteria and ABY1 showed median doubling times of 13 to 16 days, for *Cand.* Saccharimonadia and *Cand.* Gracilibacteria, these were between 22 and 25 days, respectively. *Cand.* Berkelbacteria showed the slowest growth with a median doubling time of 36 days.

We further evaluated doubling times for non-CPR taxa present in the groundwater incubations, which might serve as hosts for CPR (Fig. 5B). Specific non-CPR ASVs showed rapid growth with doubling times of 1 to 2 days, comparable to the fast-growing CPR. These ASVs were mainly found among Gammaproteobacteria (39 ASVs of Burkholderiales and Pseudomonadales), with minor contributions of Bacteroidia (8 ASVs), Alphaproteobacteria (3 ASVs), Nitrospiria (2 ASVs), and Verrucomicrobiae (1 ASV). Most ASVs, however, showed slower growth: Bacteroidia and Verrucomicrobiae had median doubling times of 5 to 8 days, while for Proteobacteria, Nitrospiria, Planctomycetes, and Acidimicrobiia, these were in the range of 11 to 16 days. The overall median doubling times were not significantly different for CPR and non-CPR (15.4 days vs. 13.5 days, Kruskal–Wallis-test, *p* = 0.494). These results suggest that, under incubation conditions that largely mimic the oligotrophic nature of the native groundwater environment (aside from the added substrates), CPR and non-CPR bacteria can exhibit comparable growth dynamics. Representatives of both groups demonstrated the capacity for rapid proliferation, indicating that CPR are not inherently constrained in their growth potential under these conditions.

### CPR and non-CPR co-occurrence patterns vary by treatment and CPR lineage

**Fig. 6:**
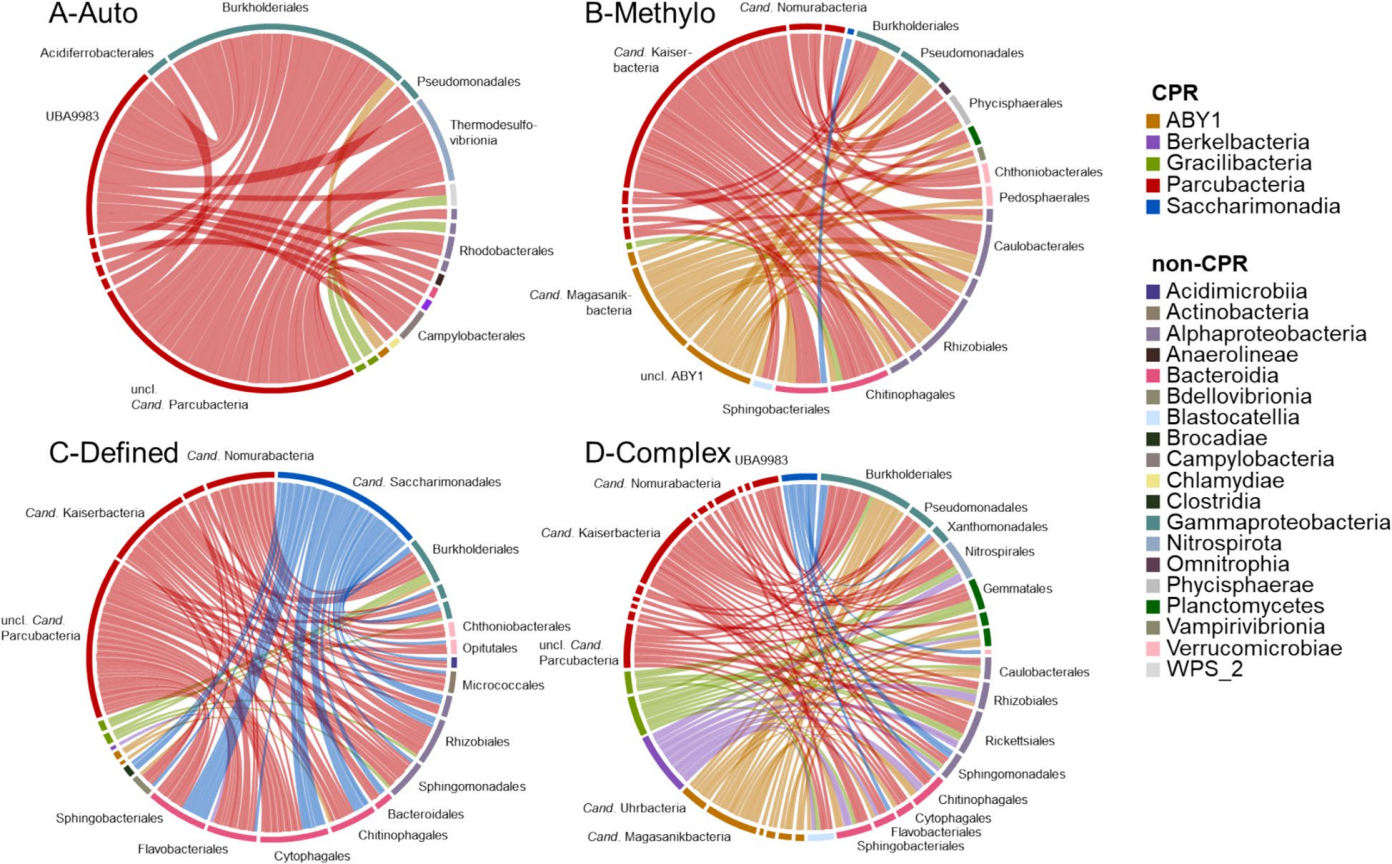
Co-occurrence of CPR and non-CPR orders. Shown are significant co-occurrences based on SparCC analysis in the **A** Auto, **B** Methylo, **C** Defined, and **D** Complex treatments. Colors of the chords inside of the circles reflect taxonomy of the co-occurring CPR, and the width of the chords corresponds to the number of co-occurrences. Only co-occurrences between CPR and non-CPR are shown. If more than 200 co-occurrences were found, only the 200 co-occurrences with the highest correlation value were selected, and only non-CPR orders with more than two co-occurrences were included for plotting. Only labels for key co-occurring orders are shown

Co-occurrence patterns between CPR and non-CPR varied distinctly across treatments. In the Auto treatments, significantly positive co-occurrences were limited in diversity, primarily involving UBA9983 of the *Cand.* Parcubacteria. Methylo treatments additionally showed frequent co-occurrences for ABY1, and Defined treatments for *Cand.* Saccharimonadia. The most diverse range of co-occurrences was observed in the Complex treatment, including all observed CPR classes except *Cand.* Roizmanbacteria (Fig. 6). Non-CPR co-occurring with CPR frequently included fast-growing organisms like Burkholderiales and Pseudomonadales, as well as members of the Bacteroidia, the latter in particular with *Cand.* Saccharimonadia. However, slower growing groups, including members of the Nitrospirota and Planctomycetes, also showed co-occurrences with CPR, including *Cand.* Gracilibacteria. These patterns suggest that CPR interactions extend to a diverse range of fast- and slow-growing lineages.

### Oxic conditions coincide with higher absolute abundance of CPR

**Fig. 7:**
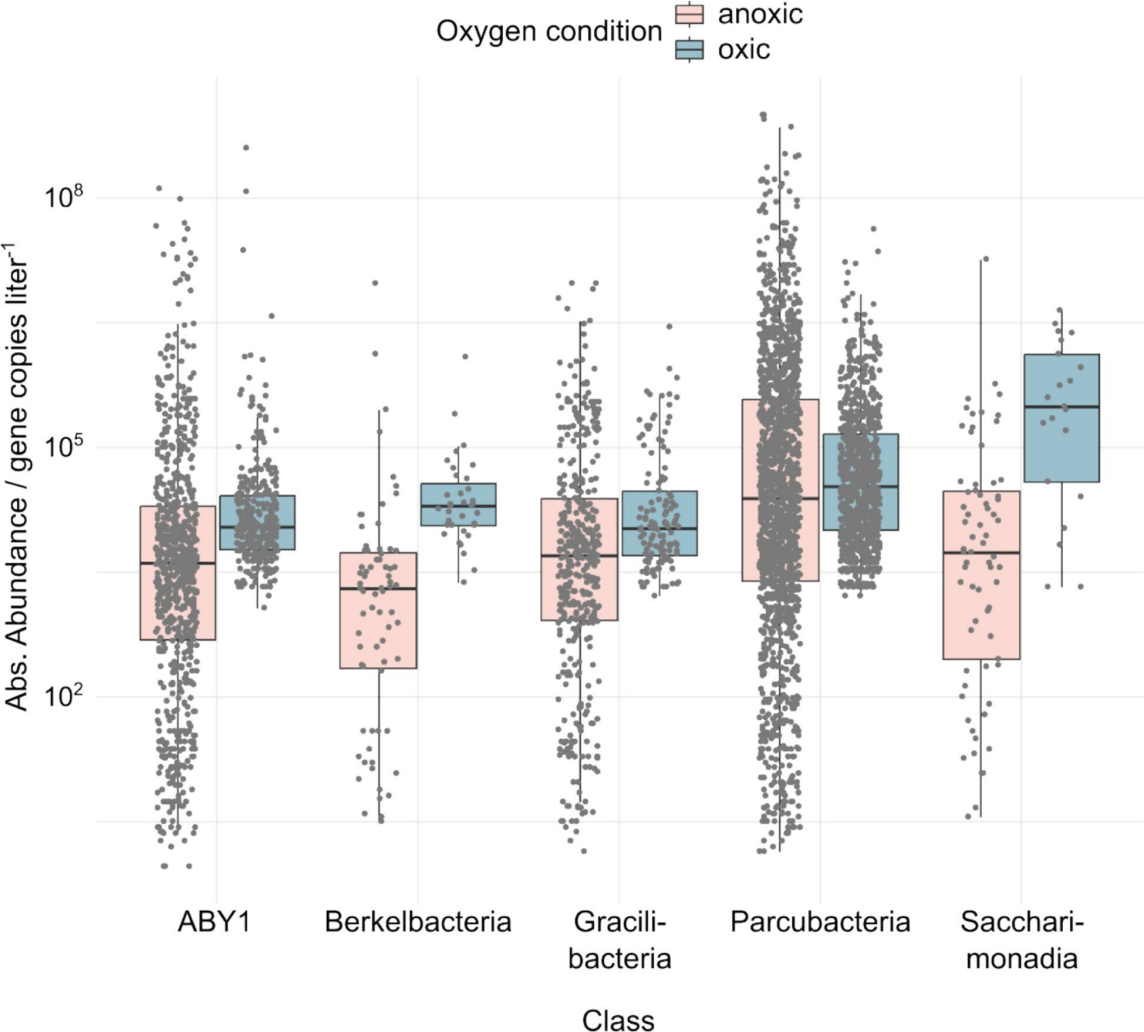
Absolute abundances of CPR ASVs in oxic and anoxic groundwater enrichments. Absolute abundances were calculated by multiplying relative abundance from 16S rRNA gene sequencing and total absolute 16S rRNA gene copy number from quantitative PCR. Dots represent individual ASVs. Boxes show median, first and third quartile, whiskers show minimum and maximum values

Despite the primarily negative correlations of ASV with oxic conditions, CPR ASVs across all five major classes observed exhibited higher median absolute abundances under oxic compared to anoxic conditions (Fig. 7). Notably, *Cand.* Berkelbacteria and *Cand.* Saccharimonadia showed median absolute abundances tenfold and 50-fold higher under oxic than under anoxic conditions, which suggested a particular preference for oxygen in these groups. Similarly, *Cand.* Parcubacteria, ABY1, and *Cand.* Gracilibacteria showed slightly (1.3- to 2.7-fold) but significantly higher median absolute abundance under oxic conditions (*p* values of 6.81 × 10^−4^, 2.08 × 10^−9^, and 2.21 × 10^−3^, respectively). This suggests that while fewer ASVs thrived under oxic conditions based on our correlation analyses, they were able to reach higher absolute abundances. However, given the nature of our dataset, which contains no pairs of oxic and anoxic samples with otherwise identical experimental conditions, we cannot exclude the possibility that other co-varying factors contributed to the observed differences. Hence, oxygen may be associated with, but not necessarily responsible for, the higher abundances of CPR observed. Even so, these patterns provided the motivation to examine more closely the potential associations between CPR and oxic conditions.

### CPR MAGs feature genes for oxygen-utilizing reactions

To determine functional traits of CPR taxa that indicate preferences for an aerobic lifestyle, we investigated the metabolic capabilities of 587 CPR MAGs previously obtained from groundwater of the Hainich CZE [11]. The selection of the same source material as used for the incubations described above ensures an optimal overlap between ASV data and MAGs. First, we classified MAG functions based on the oxic/anoxic metabolic network analysis conducted by Raymond and Segrè [49]. This analysis groups biochemical reactions into three categories: (I) oxic reactions that can only occur in the presence of oxygen or metabolites that can only be synthesized with oxygen, (II) anoxic reactions that are possible strictly without oxygen or oxygen-derived metabolites, and (III) augmented reactions, representing anaerobic metabolic processes being replaced by alternative reactions in aerobes.

**Fig. 8:**
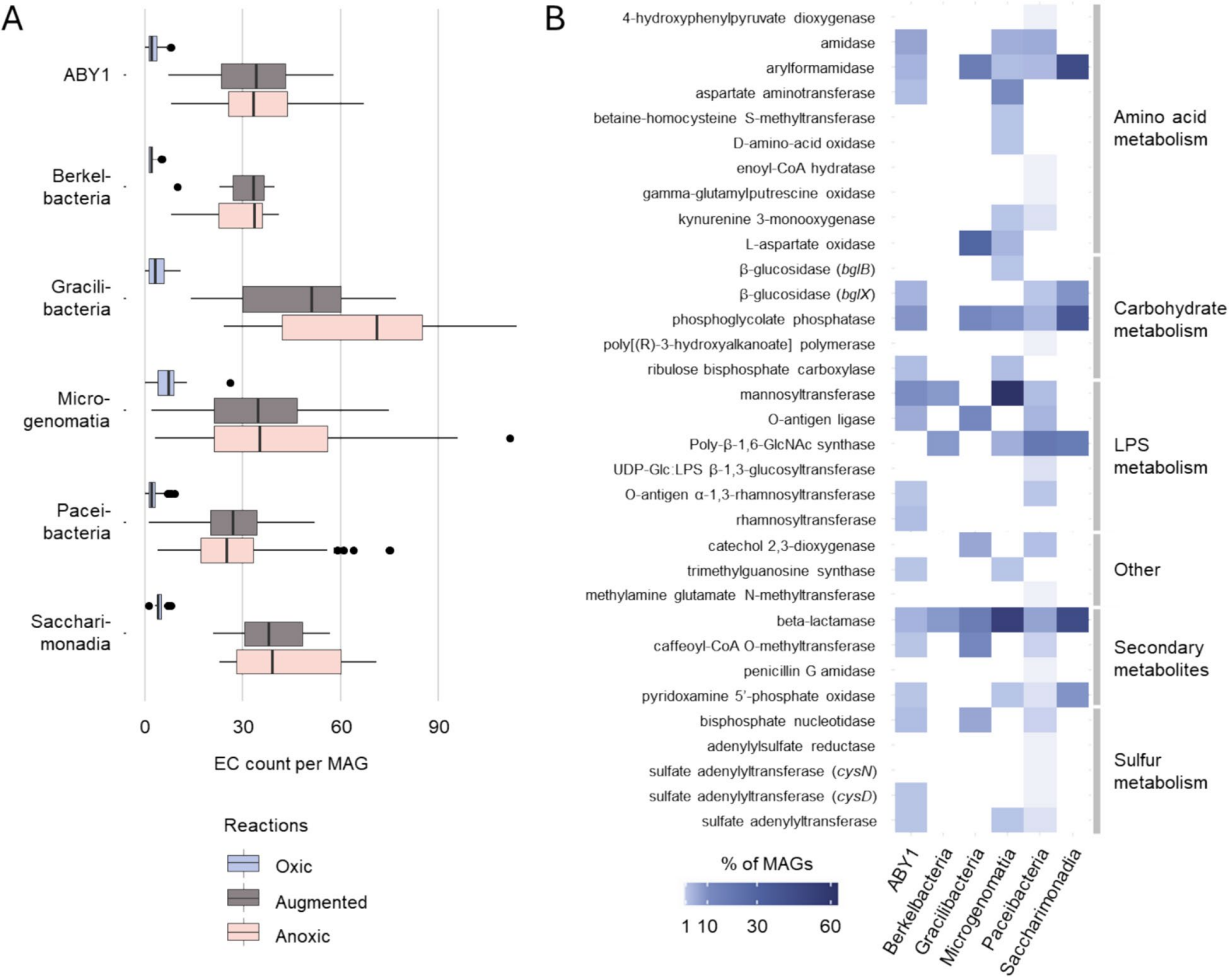
Occurrence of oxygen-dependent genes in CPR. **A** Count of EC numbers represented by the genes of each CPR MAG, grouped based on their presence in the anoxic, augmented or oxic reaction subnetwork as described in Raymond and Segrè, 2006 [49]. Boxes show median, first and third quartile, whiskers show minimum and maximum values, excluding outliers defined as exceeding 1.5 * interquartile range, which are shown as points. **B** Functional categories of genes from the oxic reaction subnetwork present in CPR. Color intensity shows the percentage of MAGs containing genes from the respective category. Data is based on functional annotation with DRAM

Surprisingly, out of the 577 CPR MAGs, 503 contained genes mapping to the oxic reaction network, with median counts of 2 to 7 genes per MAG (Fig. 8A, Table S5). As expected from anaerobes, a higher number of genes mapped to the anoxic reaction network, with median gene counts of 25 to 71 across the CPR classes. Notably, 27 to 51 genes per MAG were assigned to the augmented reaction network, suggesting that metabolic processes in CPR might be modified in adaptation to oxic conditions, enhancing their metabolic flexibility.

A deeper look into matches to the oxic reaction network revealed genes encoding enzymes that use oxygen directly for their reactions, such as oxygenases and certain oxidases. Specifically, the gene for l-aspartate oxidase, involved in nicotinate and nicotinamide metabolism as well as alanine, aspartate, and glutamate metabolism, was identified in *Cand.* Gracilibacteria (5 MAGs) and *Cand.* Microgenomatia (3 MAGs). The encoded enzyme can utilize both oxygen and fumarate to reoxidize its cofactor [55], and could therefore be a way for CPR to use oxygen. The gene of kynurenine 3-monooxygenase, active in tryptophan metabolism, was found in *Cand.* Microgenomatia (1 MAG) and *Cand.* Paceibacteria (2 MAGs). The pyridoxamine 5′-phosphate oxidase gene, which plays a role in vitamin B6 metabolism, was present across various CPR groups: *Cand.* Saccharimonadia (1 MAG), ABY1 (1 MAG), *Cand.* Microgenomatia (1 MAG), and *Cand.* Paceibacteria (2 MAGs). Furthermore, the catechol 2,3-dioxygenase gene, linked to styrene degradation, appeared in *Cand.* Paceibacteria (7 MAGs) and *Cand.* Gracilibacteria (1 MAG). In addition, one MAG of *Cand.* Paceibacteria contained the gene for 4-hydroxyphenylpyruvate dioxygenase, which participates in phenylalanine metabolism and ubiquinone biosynthesis. The d-amino-acid oxidase gene, associated with metabolic pathways of penicillin and cephalosporin biosynthesis as well as arginine and proline metabolism, was identified in one *Cand.* Microgenomatia MAG.

In general, the genes mapping to the oxic reaction network involved various functions related to amino acid metabolism, covering 81 CPR MAGs (Fig. 8B). In particular, genes for tryptophane metabolism were present across all classes except for *Cand.* Berkelbacteria, with the highest frequency of 18% of MAGs for *Cand.* Saccharimonadia. Further oxygen-related genes present in 95 MAGs were linked to biosynthesis of secondary metabolites, such as vitamin B6, flavonoids, and beta-lactam antibiotics. Such functions were present in particular in more than 50% of *Cand.* Microgenomatia and *Cand.* Saccharimonadia MAGs. A total of 180 MAGs contained oxygen-related genes affiliated with lipopolysaccharide biosynthesis (Table S5). These covered 67% of *Cand.* Microgenomatia and 27% of *Cand.* Paceibacteria MAGs. Further oxygen-related functions for carbohydrate metabolism, like phosphoglycolate phosphatase and beta-glucosidase, were present in 48 MAGs, including 45% of *Cand.* Saccharimonadia MAGs.

**Fig. 9:**
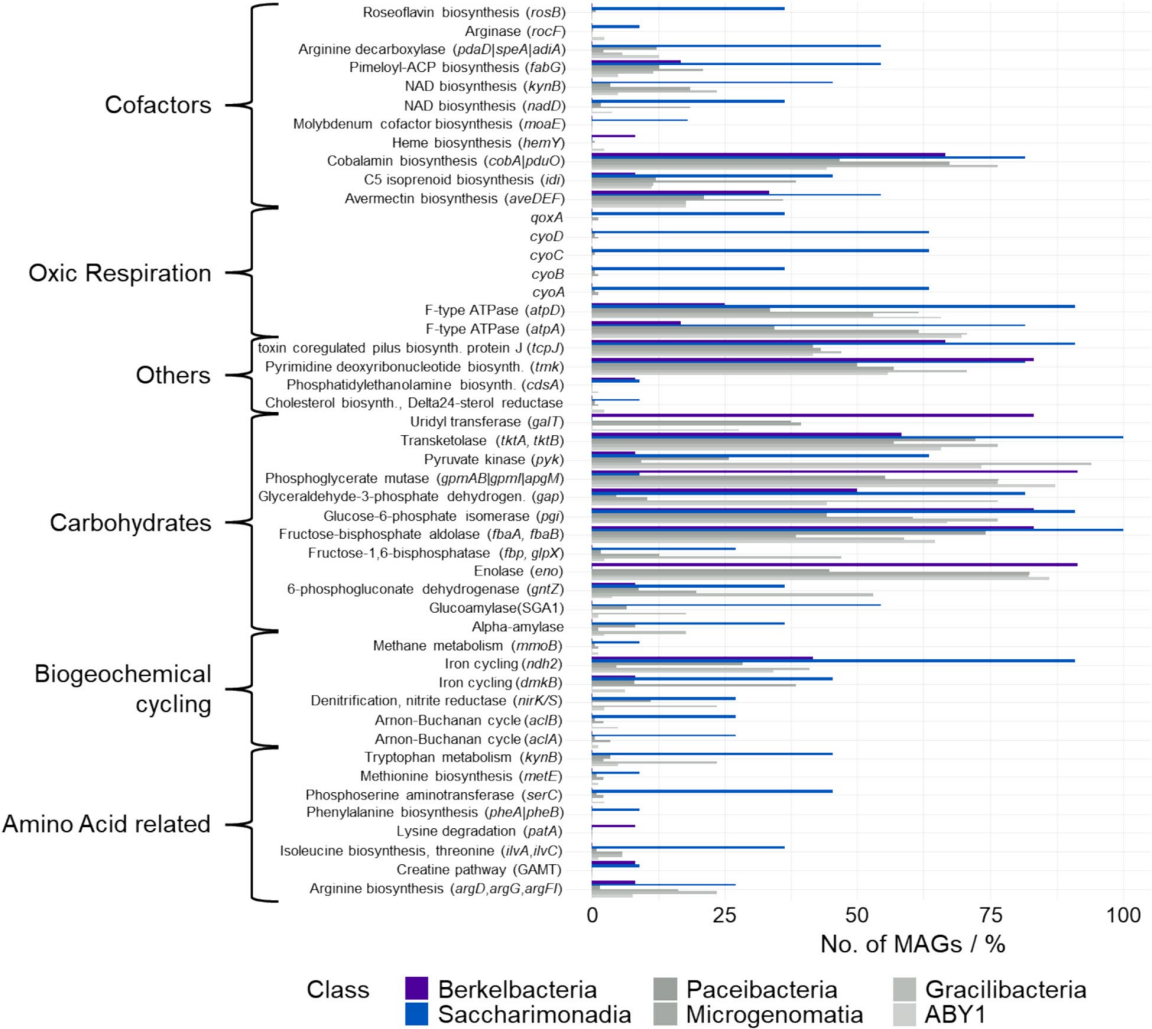
Genes significantly enriched in CPR with preference for oxic incubations. Shown are genes that are present in significantly higher frequency in MAGs of *Cand.* Berkelbacteria or *Cand.* Saccharimonadia compared to all other CPR, based on Kruskal Wallis tests (*p* < 0.05). Values given are the percentage of MAGs of the respective class containing the corresponding gene. Data is based on annotation with METABOLIC-G

In a second, complementary analysis, we focused on the genomic repertoire of the CPR classes with an observed higher abundance under oxic conditions, *Cand.* Saccharimonadia and *Cand.* Berkelbacteria. Here, we searched for functions that are significantly more common in these taxa than in other CPR. Genes for complex IV of the respiratory chain (*cyoABCD*, *qoxA*) were significantly enriched in *Cand.* Saccharimonadia, being present in 36–64% of MAGs (Fig. 9, Table S6). These were absent in most other CPR, although, interestingly, were found in a few *Cand.* Parcubateria MAGs. Genes for F-type ATPase, though present in various CPR, were likewise enriched in *Cand.* Saccharimonadia. *Cand.* Saccharimonadia MAGs also featured more functions for biosynthesis of cofactors like NAD and pyridoxal-phosphate, and secondary metabolites like terpenoids and antibiotics. Furthermore, they were enriched in genes for ATP citrate lyase (*aclAB*), nitrite reductase (*nirK*, *nirS*), and iron reduction (*ndh2*, *dmkB*). Various functions for carbohydrate metabolism were enriched in both groups, like enolase, transketolase, and aldolase (*fbaA*). *Cand.* Saccharibacteria additionally featured genes for amylases, while *Cand.* Berkelbacteria frequently possessed genes involved in nucleo-sugar biosynthesis. Lastly, *Cand.* Saccharimonadia were also enriched in various functions for amino acid biosynthesis and degradation compared to other CPR. In summary, the metabolic potential for reactions directly or indirectly dependent on oxygen is present throughout all groups of CPR investigated, and specific functions are enriched in CPR classes with a preference for oxic conditions.

## Discussion

Our study provides compelling evidence that a surprisingly broad diversity of CPR bacteria can survive and even grow under laboratory incubation conditions. Across hundreds of groundwater incubations, we detected active growth of CPR taxa from six major classes, including not only the dominant groups in situ, such as *Cand.* Parcubacteria, ABY1, and *Cand.* Microgenomatia [11], but also less prevalent groups like *Cand.* Saccharimonadia, *Cand.* Gracilibacteria, and *Cand.* Berkelbacteria. In total, we identified 1410 CPR amplicon sequence variants (ASVs), representing 74% of the CPR orders previously detected in the Hainich CZE groundwater system. While these ASVs accounted for only a fraction of the full in situ CPR diversity (up to 22,278 ASVs; [56]), their presence demonstrates that a substantial portion of this taxonomic diversity retains the capacity for growth under defined, yet artificial, laboratory conditions. This challenges assumptions about the extreme specialization and cultivation resistance of CPR taxa and offers a new window into their ecological plasticity.

As the primers used for amplicon sequencing cover more than 90% of CPR sequences in the SILVA database of five of the classes investigated (see Table S2), we believe that we cover the major part of these organisms in the incubation. One exception was the class *Cand.* Microgenomatia, of which only 0.5% of sequences were covered. This matches with the low number of ASVs (3 out of 1410 CPR ASVs) affiliated to this group. Our study therefore probably does not cover the majority of this class despite it being the third most abundant CPR class in situ [11]. The primer pair used for quantitative PCR showed a slightly lower coverage, of between 80.1 and 92.5% of sequences per class (see Table S2). Depending on the particular ASVs present, we hence cannot exclude a minor underestimation of the absolute abundances determined in our analysis. However, since our conclusions are largely based on comparisons between samples or time points, any such underestimation would be systematic and consistent across datasets. Therefore, relative differences remain robust and interpretable, mitigating the impact of primer bias on our overall findings.

Several CPR taxa exhibited clear preferences for specific treatment conditions. For instance, the Auto incubations, supplemented with thiosulfate and ammonium to promote chemolithoautotrophic growth, were particularly beneficial for the enrichment of *Cand.* Microgenomatia. For the growth of *Cand.* Saccharibacteria, a supplementation with polysaccharides in the Defined treatments was most suitable. Given the lack of metabolic potential for autotrophy in CPR [57], it is unlikely that *Cand.* Microgenomatia directly used the inorganic electron donors for autotrophic growth, and might rather be supported indirectly by the growth of other bacteria with these compounds. For *Cand.* Saccharimonadia, however, an active use of the added polysaccharides is plausible based on their reported metabolic potential, which features various glycosyl hydrolases for polysaccharide degradation [58]. This also matches with their primary occurrence in soils, seepage and groundwater wells closest to the recharge area of the Hainich CZE [59], where such plant-derived compounds would play the largest roles.

Other CPR taxa occurred more general across different treatments, like members of *Cand.* Parcubacteria (especially *Cand.* Kaiserbacteria and *Cand.* Nomurabacteria) and class ABY1. In agreement, these groups also show a highly consistent presence across the groundwater transect of the Hainich CZE, independent of groundwater age and oxygen availability [11, 59].

In groundwater incubations, CPR abundances initially decreased strongly compared to in situ levels, but often recovered after several weeks, eventually reaching values similar to those in the original groundwater. This delayed recovery has previously been attributed to the presumed slow growth of CPR [60, 61], enabling faster-growing taxa to outcompete them early in incubations. However, our data show that CPR exhibit growth rates comparable to other bacteria: most taxa, CPR and non-CPR alike, have long doubling times of several weeks, with only a few being capable of doubling within a day. These patterns likely reflect an adaptation to the oligotrophic conditions of groundwater, which select for slow-growing organisms not only in CPR, but more broadly.

The delayed growth of CPR might therefore have other reasons. Incubations conducted in oligotrophic groundwater, even when supplemented with a defined or complex substrate, still impose strong nutrient limitations. Accordingly, total bacterial abundances only increased by one or two orders of magnitude, following stagnation likely caused by nutrient limitations. The stress resulting from this starvation could make bacterial cells more susceptible to parasitism or predation by CPR [62], or could lead to increased cell lysis and metabolite release that CPR might benefit from passively. Creating such starvation conditions might hence be another key factor for enriching CPR. Thus, starvation-induced susceptibility of host organisms may be a key factor enabling CPR enrichment in laboratory incubations and could mirror ecological dynamics in situ.

Previous estimates of CPR growth derived from individual MAGs, using measures like iREP [60] and GRiD [63], suggested low growth rates for the majority of CPR, but also yielded prominent higher values for individual CPR, e.g., from *Cand.* Parcubacteria [60], similar to our observations. Unfortunately, these metagenome-derived metrics cannot easily be translated into doubling times [64]. From the few available co-cultures, growth rates for *Cand.* Saccharimonadia with Actinobacteria as hosts were reported in the range of just below 1 day to around 1 h [10, 65], matching with the fast-growing CPR found in our study.

The fast-growing *Cand.* Parcubacteria and ABY1 showed co-occurrence with various members of fast-growing Proteobacteria (Pseudomonadales, Burkholderiales), supporting the hypothesis that fast-growing hosts could benefit the growth of CPR [60, 66]. Our co-occurrence patterns furthermore endorse the recently suggested expansions of the host ranges of the epiparasitic CPR groups *Cand.* Gracilibacteria and *Cand.* Saccharimonadia [18, 20], by including Nitrospirota and Bacteroidota, respectively, as potential hosts. Bacteroidota, in particular, also co-occurred frequently with other CPR groups, which agrees with reports from previous studies [11, 66]. Bacteroidota often play roles in the degradation of complex exopolysaccharides and proteinaceous matter, traits that are shared by CPR. Hence, both groups may benefit from the conditions present in enrichments depleted in easily accessible nutrients.

**Fig. 10:**
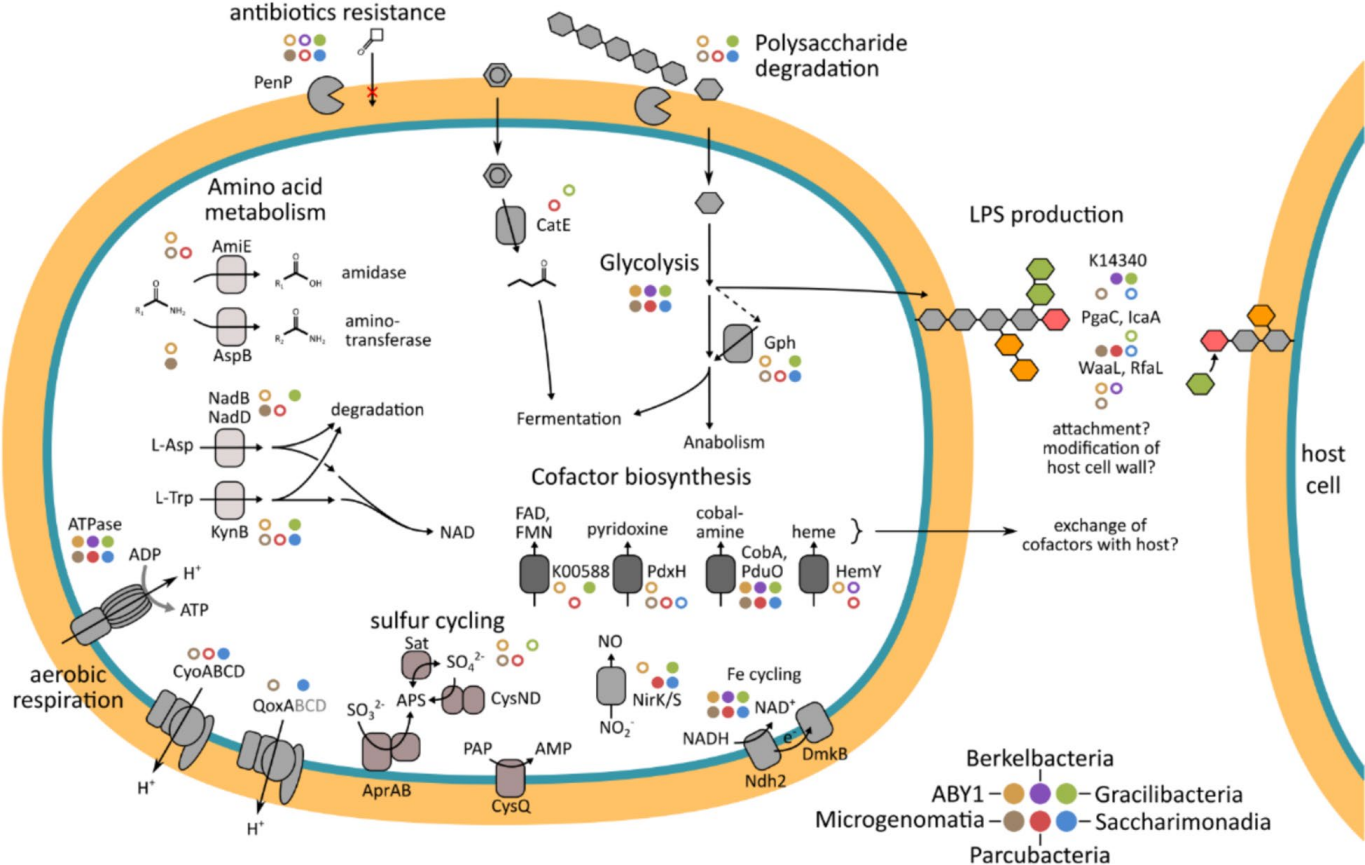
Genetic potential related to the presence of oxygen in six classes of CPR. Functions shown either depend on oxygen or oxygen-dependent metabolites, or were significantly enriched in CPR classes favoring oxic conditions. Colored circles indicate classes of CPR containing the respective functions. Empty and filled circles indicate presence in less or more than 10% of CPR MAGs, respectively

The presence of oxygen led to higher absolute abundances of CPR in the enrichments, although CPR are typically believed to have an anaerobic, fermentative metabolism [3]. In addition, while possessing complex V of the respiratory chain, required for the synthesis of ATP from proton gradients, CPR lack any other complexes, rendering them incapable of respiring oxygen for energy generation [11, 19]. Exceptions to this pattern are *Cand.* Saccharibacteria, for which the machinery for oxic respiration has been reported in surface soils [67, 68]. Our results suggest that also a substantial proportion of *Cand.* Saccharibacteria from groundwater can respire oxygen. Furthermore, the presence of cytochrome o oxidase genes in several *Cand.* Parcubacteria MAGs indicates that this trait might be shared by further CPR classes. Apart from aerobic respiration, our analysis highlighted the presence of various genes affiliated with oxygen-dependent processes across all classes of CPR investigated (Fig. 10). This included genes for enzymes using oxygen as substrate, pointing to the direct use of oxygen in CPR metabolism. An even higher number of genes affiliated with the augmented reaction subnetwork were found in CPR, indicating the widespread use of metabolites arising from directly oxygen-dependent reactions. These can be provided by the oxygen-dependent reactions CPR carry out themselves, however, given their host-associated lifestyle, can also be obtained from aerobic hosts.

Both in genes associated with the oxic reaction network and enriched in CPR preferring oxic conditions, we found genes for individual steps of different biogeochemical cycles as well as genes affiliated with the production of secondary metabolites like cofactors. Some of these, like genes for NAD and heme biosynthesis, might be tied to the oxygen-dependent metabolism of the CPR. However, their presence also indicates that CPR could provide such cofactors for other organisms. The mutualistic, rather than purely parasitic, traits of CPR might hence extend beyond metabolic handoffs in nitrogen and sulfur cycling [69]. Furthermore, the presence of genes for amino acid and carbohydrate metabolism affiliated with an oxic lifestyle points to a higher metabolic flexibility of these CPR. In the economical lifestyle of CPR, affording the costs of additional genes might be compensated for by gains from integrating oxygen-dependent processes into their metabolism. All these aspects might improve the success of CPR under oxic conditions.

## Conclusions

Here, we employed amplicon datasets from 397 groundwater enrichments to understand CPR growth dynamics and preferences under different conditions. Our findings show that a wide variety of CPR, covering six major classes, can grow in laboratory incubations, with growth rates comparable to non-CPR. This challenges assumptions of CPR as strictly slow-growing organisms. The observed group-specific responses to enrichment conditions point to distinct metabolic dependencies, with some CPR benefitting from addition of particular carbon compounds and others from stimulation of microbial processes, or induction of starvation in non-CPR bacteria.

Notably, the higher enrichment of CPR under oxic conditions, contrasting with their presumed anaerobic lifestyle, and the genomic potential for oxygen-related functions suggest that oxygen plays a more integral role in CPR metabolism than previously recognized. These results support the emerging view of CPR as metabolically flexible organisms that can benefit from the advantages of aerobic metabolism while having outsourced most of the directly oxygen-dependent reactions to their hosts. Our work hence expands the ecological and physiological understanding of CPR and provides a foundation for the development of more targeted cultivation strategies in future studies. 

## Supplementary Information

Additional file 1. Supplementary information.

Additional file 2. Tables S1–S6.

## Data Availability

Raw data from 16S rRNA gene amplicon sequencing has been deposited in the NCBI short read archive (SRA). Bioproject accessions for previously published data can be found in Table S1. Previously unpublished data can be accessed via Bioproject accession PRJNA1227287. The CPR MAGs used for this study are available at Open Science Framework (OSF) repository: https://osf.io/wq7tr/. Annotations from DRAM and intermediate result files from sparcc analysis can be found at: http://osf.io/zgh2n/.
