# Supplementary material for: Growth of candidate phyla radiation bacteria in groundwater incubations reveals widespread adaptations to oxic conditions
Source: Microbiome. 2025 Oct 30;13:224. doi: 10.1186/s40168-025-02244-1 (PMC12577380; doi:10.1186/s40168-025-02244-1)
Supplement: Supplementary file 1 — Additional file 1. Supplementary information. [file 40168_2025_2244_MOESM1_ESM.docx]

Growth of Candidate Phyla Radiation bacteria in groundwater incubations reveals widespread adaptations to oxic conditions

Ekaterine Gabashvili^1,2^, Kirsten Küsel^1,2,3^, Akbar Adjie Pratama^1,2,4,5,6^, He Wang^1^, Martin Taubert^1,2 *^

^1^Aquatic Geomicrobiology, Institute of Biodiversity, Ecology & Evolution, Friedrich Schiller University Jena, Dornburger Str. 159, Jena 07743, Germany

^2^Cluster of Excellence Balance of the Microverse, Friedrich Schiller University Jena, Jena, Germany

^3^German Center for Integrative Biodiversity Research (iDiv) Halle-Jena-Leipzig, Puschstrasse 4, 04103, Leipzig, Germany

^4^Department of Microbiology, Ohio State University, Columbus, OH, 43210, USA

^5^Center of Microbiome Science, The Ohio State University, Columbus, OH, USA

^6^National Science Foundation EMERGE Biology Integration Institute, Columbus, OH, USA

* Correspondence:

Dr. Martin Taubert

Email: [martin.taubert@uni-jena.de](mailto:martin.taubert@uni-jena.de)

Keywords: Candidate Phyla Radiation, Patescibacteria, groundwater, autotrophy, methylotrophy

# Supplementary methods

## Origin of the investigated data

The comprehensive bacterial 16S rRNA gene amplicon sequencing dataset used in our study was derived from various previously conducted incubation experiments using groundwater of the Hainich Critical Zone Exploratory [[1](#_ENREF_1)]. The original purpose of these experiments was to gain an understanding of different functional aspects of the groundwater microbiome, such as the identification of microorganisms responsible for the degradation of plant-derived compounds or microbial necromass, or the impact of primary production in the groundwater by chemolithoautotrophs. Here, we reused this data for a detailed analysis of the response of the members of the Candidate Phyla Radiation to the incubation conditions used. A part of the datasets have previously been published [[2-6](#_ENREF_2)], and information about the incubation setup and conditions used can be found in the respective articles. For the remaining, so far unpublished data, details for the experimental conditions used can be found in the following paragraphs.

## Short term incubations targeting chemolithoautotrophs

A series of incubation experiments were performed to identify suitable conditions for the enrichment of chemolithoautotrophic microorganisms and to investigate their role in community-level carbon cycling.

The first experiment aimed to test the influence of different incubation volumes and biomass enrichment strategies. Aerobic incubations were conducted with groundwater from well H41 and anaerobic incubations with groundwater from well H52 (No. 1-4 in Table S1). Three treatments were implemented in triplicate: 1) Groundwater only, including 1 L bottles with 600 ml (H41) or 1000 ml (H52) of unfiltered groundwater, 2) biomass enriched with filters, including 500 ml bottles containing 300 ml (H41) or 500 ml (H52) of groundwater plus a 0.2 µm filter with biomass from 5 L of groundwater, and 3) biomass enriched without filters, including 500 ml bottles with the same groundwater volumes as treatment 2, but biomass was detached from the filters by shaking in 10 ml groundwater before transfer. All incubations were supplemented with 5 mM thiosulfate and 200 µM ammonium, and bottles were sacrificed after 18 days of incubation.

The second experiment aimed to further assess chemolithoautotrophic enrichment under anoxic conditions, and was conducted using groundwater from well H52 only (No. 5-6 in Table S1). Two treatments were implemented in triplicate: 1) Large-volume enrichments with 8 L of groundwater in 10 L bottles, and 2) concentrated biomass enrichments with 500 ml of groundwater in 500 ml bottles with the addition of a 0.2 µm filter containing biomass from 5 L of groundwater. All treatments were supplemented with 200 µM ammonium, 2.5 mM thiosulfate, and 200 µM nitrate. Bottles were sacrificed after 7 and 14 days of incubation. As a reference, unincubated controls were prepared by filtering 5 L of H52 groundwater (in triplicate) onto 0.2 µm filters at the start of the experiment.

To investigate carbon assimilation by chemolithoautotrophs, a third anaerobic experiment was conducted with groundwater from well H52 (No. 7 in Table S1). Twelve 1 L bottles were each filled with 1 L of groundwater and amended with 200 µM ammonium, 2.5 mM thiosulfate, and 2.5 mM nitrate. Six bottles were supplemented with ^12^C-bicarbonate and six with ^13^C-bicarbonate, following the protocol described in [[3](#_ENREF_3)]. Three bottles from each isotopic treatment were sacrificed after 7 days, and the remaining bottles after 41 days of incubation.

## Long term incubations targeting chemolithoautotrophs

To enrich distinct groups of chemolithoautotrophic microorganisms and assess their potential interactions with co-dependent community members, groundwater incubations were conducted using samples from wells H41 and H52 (No. 8-11 in Table S1). Aerobic conditions were maintained for incubations using groundwater from H41, while anaerobic conditions were used for H52-derived incubations.

Each treatment was designed to selectively stimulate specific functional groups. For aerobic incubations (H41), bottles were supplemented either with 200 µM ammonium to target nitrifiers, or with 15 µM ammonium and 2.5 mM thiosulfate to promote the growth of sulfur-oxidizing bacteria. Anaerobic incubations (H52) received either 200 µM ammonium, 120 µM nitrate, and 50 µM nitrite to stimulate anaerobic ammonia-oxidizing organisms, or a combination of 200 µM ammonium, 2.5 mM thiosulfate, and 2.5 mM nitrate to enrich for anaerobic sulfur oxidizers.

All incubations were carried out in 250 ml serum bottles sealed with butyl rubber stoppers, each containing 150 ml of groundwater. Incubations were performed in triplicate per condition. Over the course of the experiment, 10 ml samples were collected from each bottle at seven time points: days 69, 76, 83, 194, 208, 518, and 1006.

## Incubations with leaf leachate

To simulate the input of surface-derived, plant-based organic matter and assess the long-term response of the groundwater microbiome, aerobic incubations were set up using groundwater from well H41 (No. 15-16 in Table S1). These incubations aimed to investigate the impact of varying concentrations of leaf leachate on microbial community dynamics.

Incubations were conducted in 10 L glass bottles, each filled with groundwater and amended with different volumes of leaf leachate to achieve final concentrations of 10%, 1%, and 0.1% (corresponding to 1000 mL, 100 mL, and 10 mL of leachate, respectively), along with unamended controls. All treatments were prepared in duplicate, and the final volume of each bottle was adjusted to 10 L with groundwater. After 1059 days of incubation, 50 ml samples were collected from each bottle for downstream analysis.

## Incubations with soil seepage

To assess the potential influence of surface-derived soil inputs on the groundwater microbiome, aerobic incubations were performed using groundwater from well H41 (No. 17-19 in Table S1). This experiment was designed to compare microbial community responses to the addition of soil seepage in combination with enriched indigenous groundwater biomass.

A total of eighteen 120 ml serum bottles were prepared, each containing 60 ml of groundwater. All bottles received enriched microbial biomass obtained from filtration of 220 L of groundwater onto glass fiber filters (0.3 µm), which were subsequently transferred into the bottles. To simulate surface input, 5 ml of soil seepage was added to twelve of the bottles, while the remaining six served as controls without seepage addition. All bottles were incubated under aerobic conditions. The six control bottles and six seepage-amended bottles were sacrificed after 21 days of incubation. The remaining six seepage-amended bottles were sacrificed after 28 days. Additionally, unincubated samples from groundwater (2x 220 L on 0.3 µm pore size glass fiber filters) and of leachate (3x 5 ml on 0.2 µm pore size polyethersulfone membrane supor® filters, PALL Corporation, Michigan, USA) were taken.

## Incubations with Pseudomonas cells

To evaluate the feasibility of tracking introduced microbial cells in groundwater for future co-cultivation experiments, aerobic incubations were performed using groundwater from well H41 (No. 22 in Table S1). The goal was to assess detectability and persistence of deuterium-labeled cells within a natural microbial background.

Eight 1 L glass bottles were filled with 900 ml of groundwater and supplemented with 10 ml R2A medium [[7](#_ENREF_7)] to stimulate microbial growth and increase cell numbers. These pre-incubations were carried out for 5 days under aerobic conditions. Following this enrichment phase, deuterium-labeled cells of a groundwater isolate (*Pseudomonas* sp.) were added to the bottles at defined mixing ratios of 1:1, 1:9, 1:19, and 1:99 (*Pseudomonas* cells to groundwater cells), with each treatment prepared in duplicate. Bottles were further incubated for 45 days before 10 ml samples were collected from each for downstream analysis.

## Short term incubations targeting methylotrophs

To assess the response of groundwater methylotrophs to the addition of one-carbon substrates, aerobic incubations were set up using groundwater from well H41 (No. 27-28 in Table S1). The experiment aimed to evaluate substrate-specific microbial community dynamics following amendment with methylamine or methanol.

A total of eighteen 1 L bottles were prepared, each filled with 750 ml of groundwater. Six bottles were amended with 2 mM methylamine, and twelve bottles with 1 mM methanol. All incubations were performed under aerobic conditions. Methylamine-amended bottles were sacrificed after 14 days. Methanol-amended bottles were sacrificed at two time points: after 7 days and 39 days of incubation.

## Long term incubations targeting methylotrophs

To enrich methylotrophic microorganisms and explore their interactions with dependent community members, aerobic incubations were conducted using groundwater from well H41 (No. 29 in Table S1). The incubations were designed to stimulate methylotroph activity through the addition of a single-carbon substrate.

Triplicate 1 L bottles were filled with 750 ml of groundwater and supplemented with 2 mM methylamine. Incubations were carried out under aerobic conditions. During the course of the experiment, 50 ml samples were collected from each bottle at regular intervals, initially every 1–2 weeks, and subsequently every 1–2 months. To maintain enrichment activity and extend the incubation, cultures were passaged approximately every six months by transferring 50 ml from each bottle into a new bottle containing 700 ml of sterile (0.1 µm filtered) groundwater, followed by repetition of the sampling regime outlined above.

# Supplementary Figures


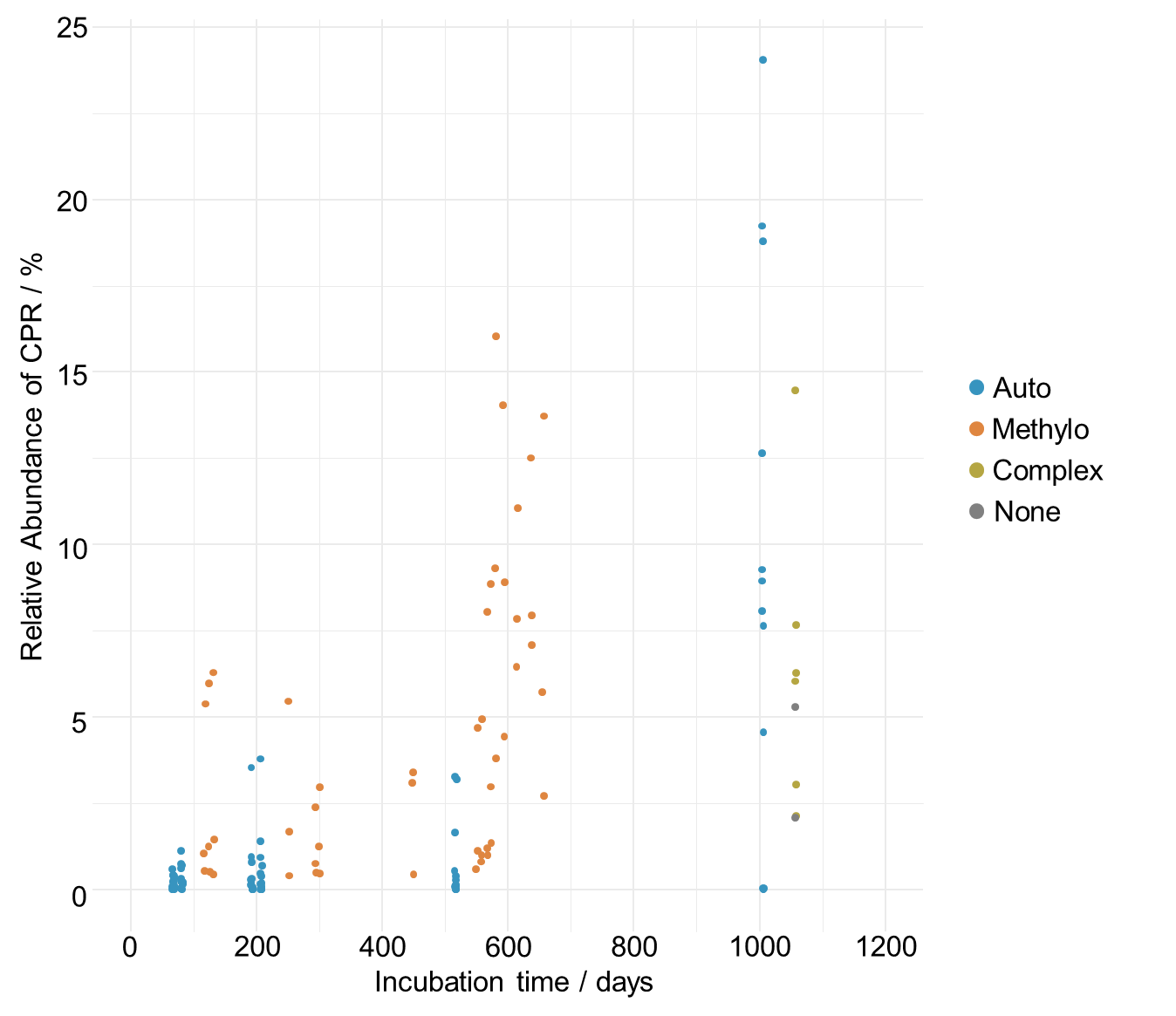

Figure S1: Abundance of CPR in long term enrichments. Only data from experiments running for more than 1 year are shown. Dots represent the relative abundances of CPR based on 16S rRNA gene amplicon sequencing in individual samples from long term enrichments with up to 3 years of incubation time. Blue dots represent samples under conditions stimulating chemolithoautotrophs (auto treatment), orange dots represent samples under conditions stimulating methylotrophs (methylo treatment), beige dots represent samples where leaf leachate was added to groundwater (complex treatment) and grey dots represent two samples of untreated groundwater incubated for 1059 days.

Figure S2: Averaged community compositions in groundwater incubations. Data is based on relative abundances of bacterial phyla from 16S rRNA gene amplicon sequencing. Bars represent average of all samples per treatment. Start includes samples from the start of incubation experiments; Auto and methylo include samples from enrichments with conditions favoring chemolithoautotrophs and methylotrophs, respectively. Defined and Complex include samples from enrichments with conditions favoring heterotrophs by adding chemically defined or complex carbon sources, respectively.


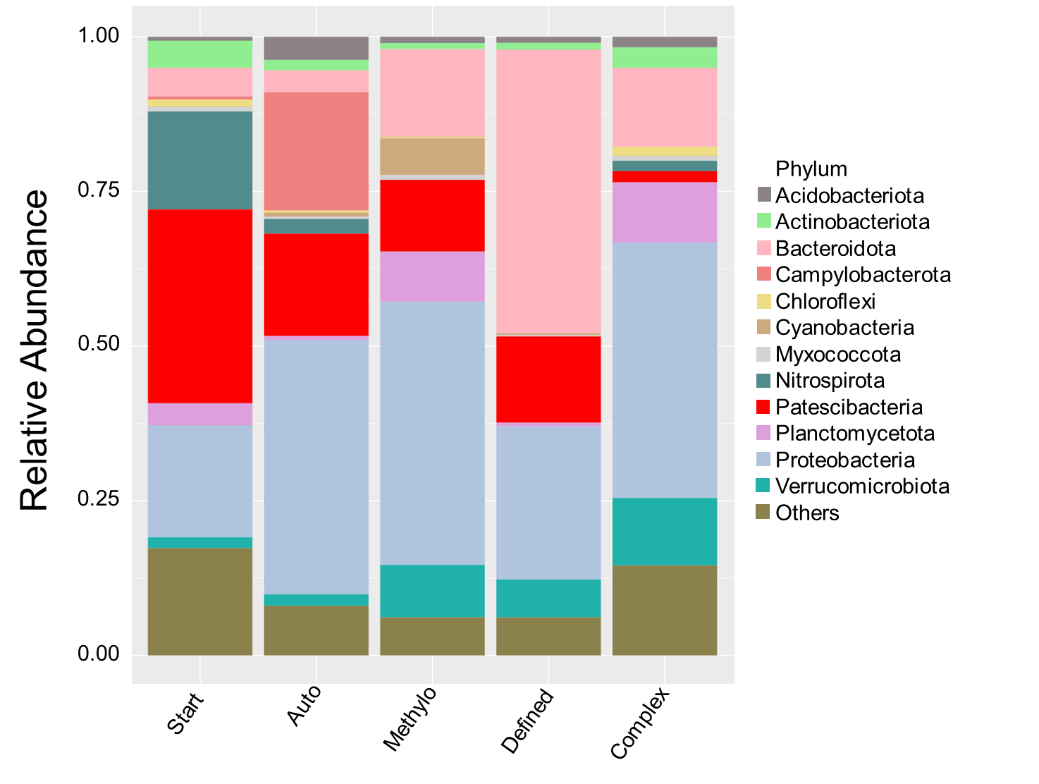


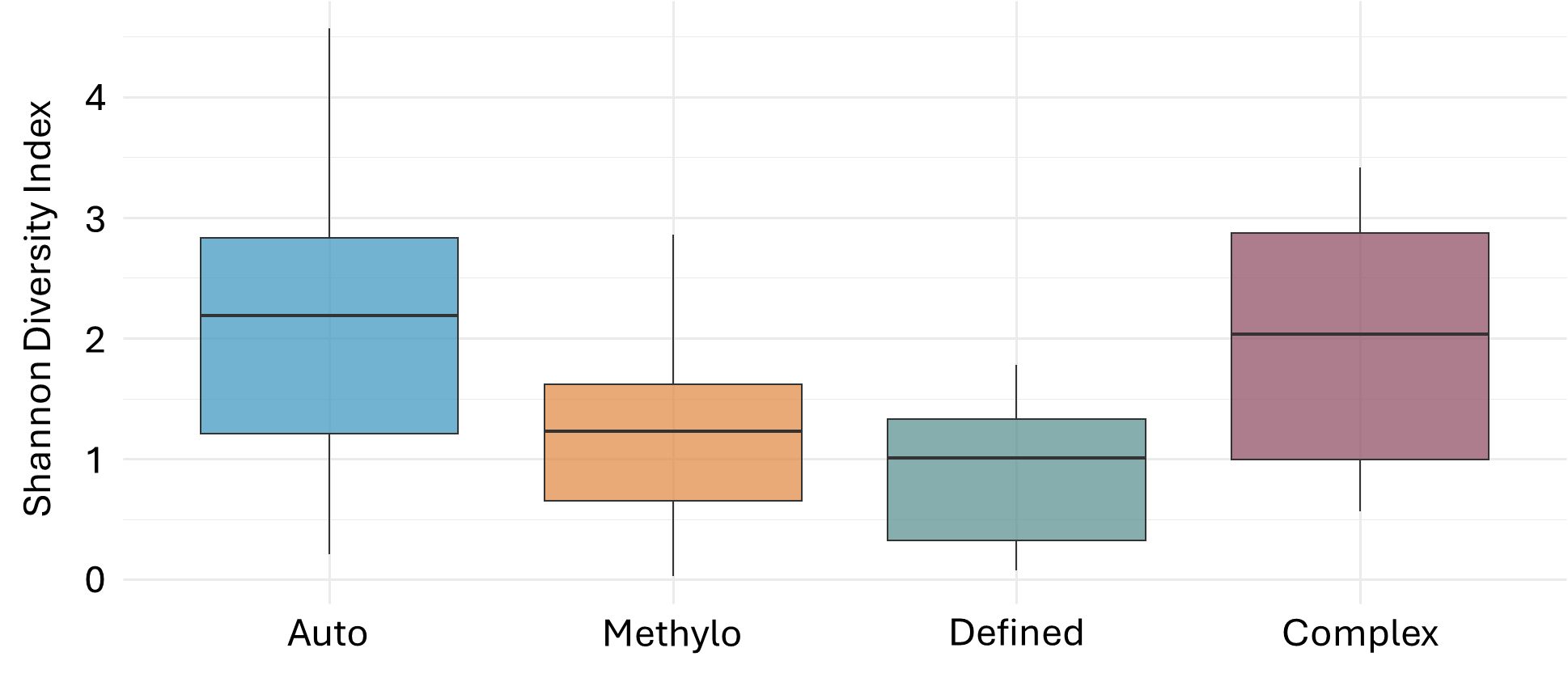


Figure S3: Shannon diversity of CPR subpopulations per treatment. Shannon diversity was calculated on a subset of the 16S rRNA gene sequencing relative abundance data filtered to only contain CPR ASVs. Boxes show median, first and third quartile, and whiskers show minimum and maximum values.

# Supplementary Table legends

Table S1: Overview of samples from groundwater incubation experiments. Rows depict sets of incubations with specific conditions. Given are the treatment the set was assigned to, the well the groundwater used for the incubations was derived from, whether the incubations were conducted under oxic or anoxic conditions, a summary of the supplements added at the start of the incubations, the volumes of the bottles used / the groundwater volume used in the incubations, the number of samples included in the set, the incubation times at which samples were taken and the number of time points sampled, the reference under which the data was originally published (if applicable), and the BioProject accession under which the raw data was deposited at NCBI.

Table S2: Coverage of primer pairs employed in the study on CPR classes. Shown is the coverage of the primer pair Bakt341F/Bakt785R used for amplicon sequencing and Bac8Fmod/Bac338Rabc used for quantitative PCR based on the CPR sequences present in the SILVA SSU r138.2 database.

Table S3: Corrected p-values and Spearman’s correlation coefficients between CPR ASVs and supplements. Rows correspond to significant correlations between individual ASVs and supplement presence. Columns show ASV number, taxonomic affiliation of the ASV, the correlating supplement, the Bonferroni-adjusted p-value and the Spearman’s rank correlation coefficient. Only correlations with coefficients >0.3 or <-0.3 and p-values below 0.001 are shown.

Table S4: Corrected p-values and log2-fold changes (lfc) of CPR ASVs and supplements. Rows correspond to significant correlations between individual ASVs and supplement presence. Columns show ASV number, ASV class, the correlating supplement, the Holm’s-adjusted p-value and the log2-fold changes in abundance of the ASV under the respective conditions. Only correlations with p-values below 0.05 are shown.

Table S5: Presence of oxygen-dependent genes in CPR MAGs. The genes shown were derived from the oxic reaction subnetwork, as described previously [[8](#_ENREF_8)]. Given are the KEGG Orthology (KO) number of the gene, the gene abbreviation, product name, EC number, the metabolic category the genes is assigned to, the category the gene was placed in (see Figure 7B in the main manuscript), as well as the number of MAGs and percentage of MAGs featuring this gene for six classes of CPR.

Table S6: Genes significantly enriched in CPR with preference for oxic incubations. Given is the functional category of the genes (see Figure 8 in the main manuscript), the gene abbreviation(s), the gene’s function or product name, the affiliated Kegg Orthology (KO) numbers, as well as the number of MAGs and percentage of MAGs featuring this gene for six classes of CPR. Bold text indicates that the respective genes are significantly enriched in the *Cand.* Saccharimonadia or *Cand.* Berkelbacteria which showed preferences for oxic incubations.

# References

1. Küsel K, Totsche KU, Trumbore SE, Lehmann R, Steinhäuser C, Herrmann M. How deep can surface signals be traced in the Critical Zone? Merging biodiversity with biogeochemistry research in a Central German Muschelkalk landscape. Front Earth Sci. 2016;4:1-18.

2. Geesink P, Taubert M, Jehmlich N, von Bergen M, Küsel K. Bacterial necromass is rapidly metabolized by heterotrophic bacteria and supports multiple trophic levels of the groundwater microbiome. Microbiol Spectr. 2022;10:e00437-00422.

3. Heinze BM, Küsel K, Jehmlich N, von Bergen M, Taubert M. Several trait-based survival strategies enable S-oxidizers to dominate primary production in groundwater. In preparation.

4. Taubert M, Overholt WA, Heinze BM, Matanfack GA, Houhou R, Jehmlich N, et al. Bolstering fitness via CO_2_ fixation and organic carbon uptake: mixotrophs in modern groundwater. ISME J. 2022;16:1153-1162.

5. Taubert M, Stähly J, Kolb S, Küsel K. Divergent microbial communities in groundwater and overlying soils exhibit functional redundancy for plant-polysaccharide degradation. Plos One. 2019;14.

6. Taubert M, Stöckel S, Geesink P, Girnus S, Jehmlich N, von Bergen M, et al. Tracking active groundwater microbes with D_2_O labelling to understand their ecosystem function. Environ Microbiol. 2018;20:369-384.

7. Reasoner DJ, Geldreich EE. A new medium for the enumeration and subculture of Bacteria from potable water. Appl Environ Microb. 1985;49:1-7.

8. Raymond J, Segrè D. The effect of oxygen on biochemical networks and the evolution of complex life. Science. 2006;311:1764-1767.
